# Modulating Perovskite Surface Energetics Through Tuneable Ferrocene Interlayers for High‐Performance Perovskite Solar Cells

**DOI:** 10.1002/anie.202424041

**Published:** 2025-01-26

**Authors:** Francesco Vanin, William D. J. Tremlett, Danpeng Gao, Qi Liu, Bo Li, Shuai Li, Jianqiu Gong, Xin Wu, Zhen Li, Ryan K. Brown, Liangchen Qian, Chunlei Zhang, Xianglang Sun, Xintong Li, Xiao Cheng Zeng, Zonglong Zhu, Nicholas J. Long

**Affiliations:** ^1^ Department of Chemistry City University of Hong Kong Kowloon 999077 Hong Kong; ^2^ Department of Chemistry Imperial College London MSRH Building, White City Campus W12 0BZ London UK; ^3^ Department of Materials Science & Engineering City University of Hong Kong Kowloon 999077 Hong Kong

**Keywords:** perovskite solar cells, energetic modulation, ferrocene interlayers, structure-function relations

## Abstract

Achieving rational control over chemical and energetic properties at the perovskite/electron transport layer (ETL) interface is crucial for realizing highly efficient and stable next‐generation inverted perovskite solar cells (PSCs). To address this, we developed multifunctional ferrocene (Fc)‐based interlayers engineered to exhibit adjustable passivating and electrochemical characteristics. These interlayers are designed to reduce non‐radiative recombination, and to modulate the work function (WF) and uniformity of the perovskite surface, thereby enhancing device performance. The key role played by the highest occupied molecular orbital energies (*E*_HOMO_) of the Fc compounds relative to the perovskite valance band maximum (*E*_VBM_) is revealed. This relationship is pivotal in controlling band bending and optimizing charge extraction. Notably, the conformationally flexible and more easily oxidized ferrocenyl‐bis‐furyl‐2‐carboxylate (**2**) is found to more effectively bind with undercoordinated Pb^2+^ surface sites and modulate interfacial energetics, resulting in inverted PSCs achieving champion efficiencies of 25.16 %. These cells also displayed excellent stability, retaining >92 % of the initial efficiency after 1,000 h of maximum power point operation at 65 °C. By correlating the broadly tunable Fc‐*E*_HOMO_ with a decreased and homogenized perovskite surface WF, our work advances our understanding of Fc‐based interlayers and opens new pathways for their application in high‐efficiency solar technologies.

## Graphical abstract

Multifunctional ferrocene (Fc)‐based interlayers have been developed to exhibit adjustable passivating and electrochemical characteristics within inverted perovskite solar cells (PSCs). The key role played by the highest occupied molecular orbital energies (*E*_HOMO_) of the Fc compounds relative to the perovskite valence band maximum (*E*_VBM_) is revealed, and this relationship is pivotal in controlling band bending and optimizing charge extraction.

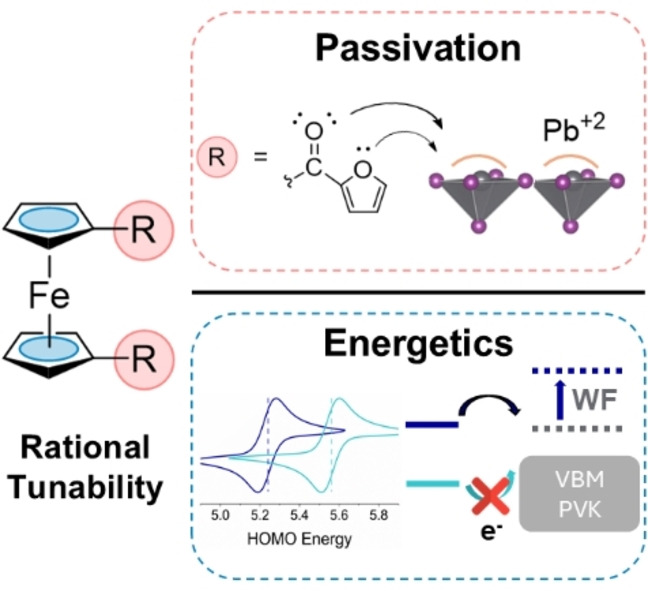

## Introduction

Perovskite solar cells (PSCs) have attracted significant research interest in the past decade primarily due to the impressive power conversion efficiencies (PCE) attainable using cost‐effective solution‐processed active layers.[^1^, ^2^, ^3^] PSCs fabricated using inverted (p‐i‐n) architectures can achieve record PCEs above 26 % and have several benefits over their conventional (n‐i‐p) counterparts, including thermal stability, large‐area processability, and compatibility with multi‐junction structures.[^4^, ^5^, ^6^] Defect‐induced non‐radiative surface recombination and energy‐level misalignment at the perovskite/electron transport layer (ETL) interface, however, remain the largest contributors to open‐circuit voltage (*V*_oc_) and fill factor (FF) losses.[^7^, ^8^] For this reason, advances in efficiency and stability have largely focused on passivating surface defects and altering interfacial energetics.[^9^, ^10^, ^11^, ^12^]

Key recent developments have revealed the importance of two crucial but previously overlooked factors limiting PSC efficiency, surface work function (WF) and WF homogeneity.^[13]^ Generally, a decrease in surface work function is observed to induce band bending and reduce minority‐carrier concentration (holes) at the interface, thus boosting charge extraction and decreasing non‐radiative recombination.^[14]^ Likewise, a concomitant homogenization of surface potential distribution ensures even and effective charge extraction. The use of organic diammonium ligands[^15^, ^16^, ^17^, ^18^] has proven effective in this regard, although surface reactions,^[19]^ borane passivation^[20]^ and 3D/3D heterojunction^[21]^ strategies have also been reported. However, a strategy to directly control perovskite surface WF properties through rational synthetic tuning, whilst simultaneously enabling robust chemical passivation is still lacking.

Organometallic compounds based on the ferrocene (Fc) framework offer several features uniquely suited to addressing this gap in the literature, including a broad library of accessible side groups and a highly tunable electrochemical potential (*E*_HOMO_) through substitution with electron‐donating and withdrawing groups.[^22^, ^23^, ^24^] Fc‐based interlayers have repeatably been shown to passivate defects and decrease surface WF, however, PSC research has focused primarily on optimizing surface binding with little attention to the electrochemical potentials of the molecules involved.[^12^, ^25^, ^26^, ^27^, ^28^, ^29^, ^30^, ^31^, ^32^] Fc molecules within only a narrow *E*_HOMO_ range (approx. 300 mV) have been studied to date, and the correlation between *E*_HOMO_ and the change in surface WF (Figure S1) has gone completely unnoticed.

**Figure 1:**
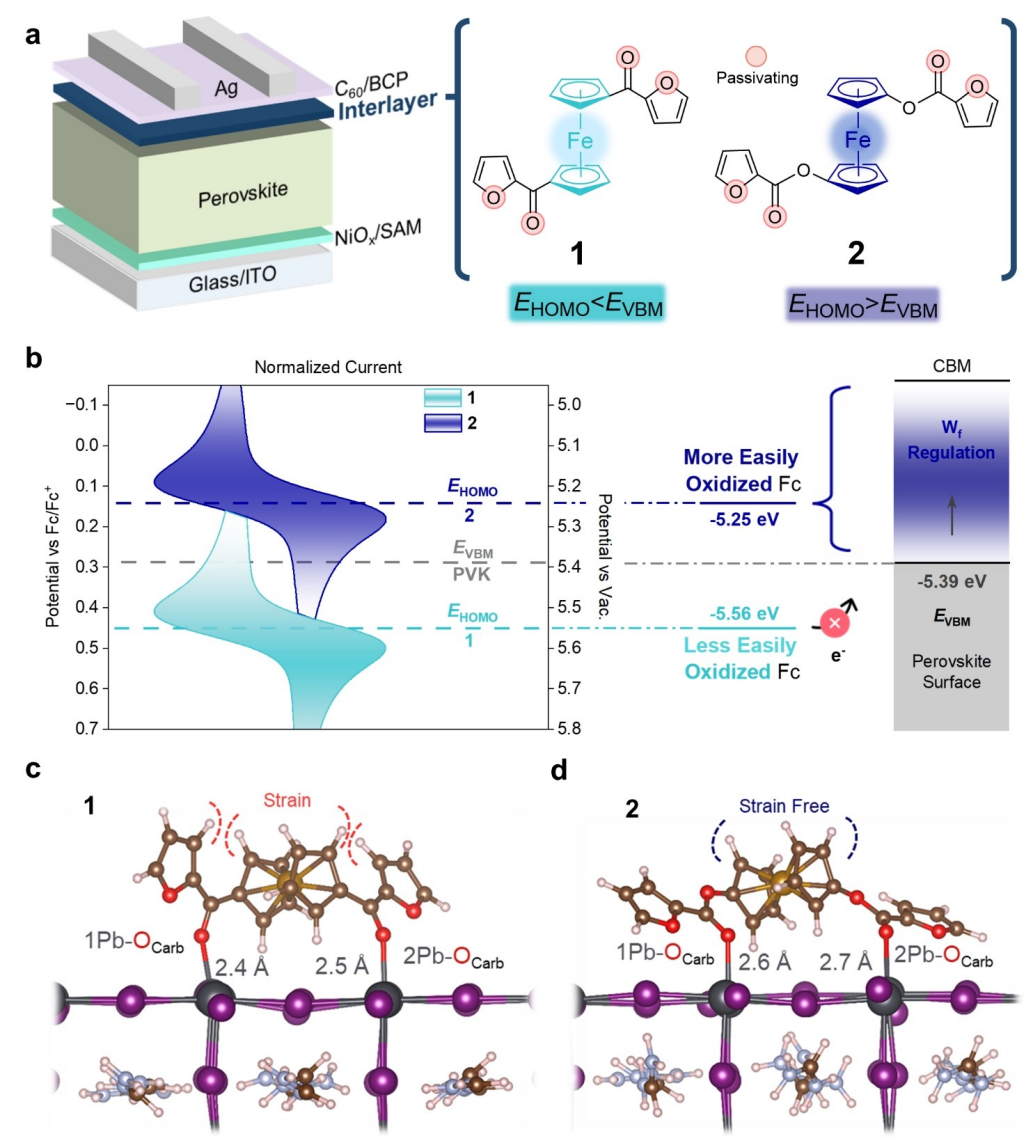
Synthetically tailoring surface properties. (a) Schematic illustrations of the inverted PSC architecture featuring **1** and **2** interlayers, key molecular design properties are highlighted. (b) Cyclic voltammetry measurements and calculated *E*_HOMO_ levels of **1** and **2** relative to the perovskite E_VBM_ alongside a sketch for the proposed WF regulation mechanism. (c) and (d) Calculated surface coordination geometries and computed molecular binding energies of **1** and **2** in the anti‐conformation with undercoordinated Pb^2+^ centers on the (001) PbI_2_ terminated perovskite surface.

Aiming to elucidate structure‐function relationships in p‐i‐n PSCs featuring Fc‐based interlayers, we investigated the effects of passivation and electrochemical potential on PSC performance and stability using two Fc‐derived compounds, ferrocenyl‐bis‐furyl‐2‐ketone (**1**) and ferrocenyl‐bis‐furyl‐2‐carboxylate (**2**) displayed in Figure 1a. These compounds were designed to have similar passivating properties through the carbonyl and heterocycle moieties, while differing significantly in electrochemical potential, having *E*_HOMO_ levels directly below (**1**) and above (**2**) the perovskite valence band maximum (*E*_VBM_).[^30^, ^33^] Both molecules successfully passivated undercoordinated Pb^2+^ states by adopting bidentate surface binding modes, however, only the more easily oxidized **2** could alter the surface WF, resulting in favorable band bending and improving the *V*_oc_ and FF of the resulting devices. Champion PSCs achieved PCEs of 25.16 % and 23.87 % after treatment with compounds **2** and **1**, respectively, maintaining >87 % and >92 % of their initial efficiency after 1,000 hours of continued maximum‐power point (MPP) operation at 65 °C. Our results elucidate a novel concept for rationally tailoring the electronic surface properties of perovskite layers using synthetically accessible Fc‐based compounds.

## Results and Discussion

We began by carefully analyzing literature reports involving organometallic compounds deposited onto perovskite layers to determine whether the unanimously observed change in surface WF following interlayer deposition was correlated with the *E*_HOMO_ of the metallocene used (Figure S1 and Table S1). Considering the results were obtained on different perovskite compositions, using different measurement techniques with compounds varying significantly in structure, we concluded that changes in surface WF showed a reasonable dependence on the compound's *E*_HOMO_ energy. Aiming to prove the synthetic accessibility to substituted Fc molecules having both strongly passivating moieties and tunable *E*_HOMO_ levels, compounds **1** and **2** were synthesized to feature similar surface binding carboxyl and furyl groups,[^34^, ^35^, ^36^] whilst differing significantly (320 mV) in electrochemical potential (Figure 1a and b). Synthetic, structural, and electrochemical characterization of **1** and **2** are presented in Figures S2–S15 and Tables S2 and S3 with further detail provided in **Notes** **S1** and **S2**. Furyl derivatives were chosen over previously reported thiophene‐based substituents to enable stronger bidentate binding through the strongly coordinating lone pairs on the oxygen atoms,[^12^, ^30^] whilst ketone (**1**) and ester (**2**) linkers were chosen for their influence on Fc's electrochemical potential.[^30^, ^33^] To accurately determine *E*_HOMO_ positions relative to the perovskite *E*_VBM_, the redox potentials measured by cyclic voltammetry (CV) were converted to the vacuum scale (eV; see **Note** **S1**), affording energies of −5.56 eV and −5.24 eV for **1** and **2**, respectively. These values are well below and above the perovskite *E*_VBM_ of −5.39 eV measured using ultraviolet photoelectron spectroscopy (UPS; Figure S16), as shown in Figure 1b.

If the change in perovskite surface WF were related to the *E*_HOMO_ of the Fc‐based passivator, no WF change is expected for **1** given that the *E*_HOMO_ lies approx. 300 meV below any previously employed Fc interlayer and 110 meV below the perovskite *E*_VBM_. By contrast, **2** is expected to decrease the perovskite surface WF by a value consistent with previous reports (60–160 meV) of Fc molecules with similar *E*_HOMO_ levels, such as our previously reported FcTc_2_ compound (Figure S1).[^12^, ^29^, ^30^, ^37^] However, given the proposed dual function of these molecules, chemical and energetic effects must be deconvoluted to gain a clear understanding of the system.

To assess the passivating surface interactions enabled by the ester (**2**) and ketone (**1**) substituents, density functional theory (DFT) calculations were performed (Figures 1c and d). The (001) facet of the PbI_2_‐terminated FAPbI_3_ perovskite surface was chosen as a representative model of our system due to the low surface energy and strong binding with Fc‐based molecules.[^30^, ^38^] As the cyclopentadienyl (Cp) rings are known to have low activation energies for ring rotation about the Fe^2+^ center, calculations were performed on both syn‐ and anti‐conformations of **1** and **2** (Figure S17).^[39]^ DFT calculations revealed that oxygen atoms with lone pairs on both furan and ketone moieties interact strongly with adjacent undercoordinated Pb^2+^ ions, preferentially adopting bidentate binding modes in both syn‐ and anti‐conformations (key bond lengths are collected in Table S4). Bidentate perovskite binding in disubstituted ferrocenes has recently been shown to correlate positively with device performance.[^25^, ^26^] Interactions between Pb^2+^ and furan oxygens (Pb‐O_Furan_) were only observed in syn‐conformations with bond lengths calculated at 2.84 Å and 3.00 Å for **1** and **2**, respectively, considerably longer than the respective carbonyl (Pb‐O_Carb_) bond lengths at 2.50 and 2.64 Å. This is consistent with the lower availability of lone pair donation on the aromatic furyl oxygen.^[40]^ Additionally, binding energy (Eb) calculations were performed on **1** and **2** in the anti‐configuration. A more negative Eb was obtained for **2** (−4.44 eV) than its ketone analog **1** (−0.92 eV). This observation is attributed to the greater conformational freedom enabled by the bridging oxygen atom in the ester group of **2**, allowing the furan rings to lie parallel with the perovskite surface and avoiding the steric clash with the Cp rings observed in **1**.

**Figure 2:**
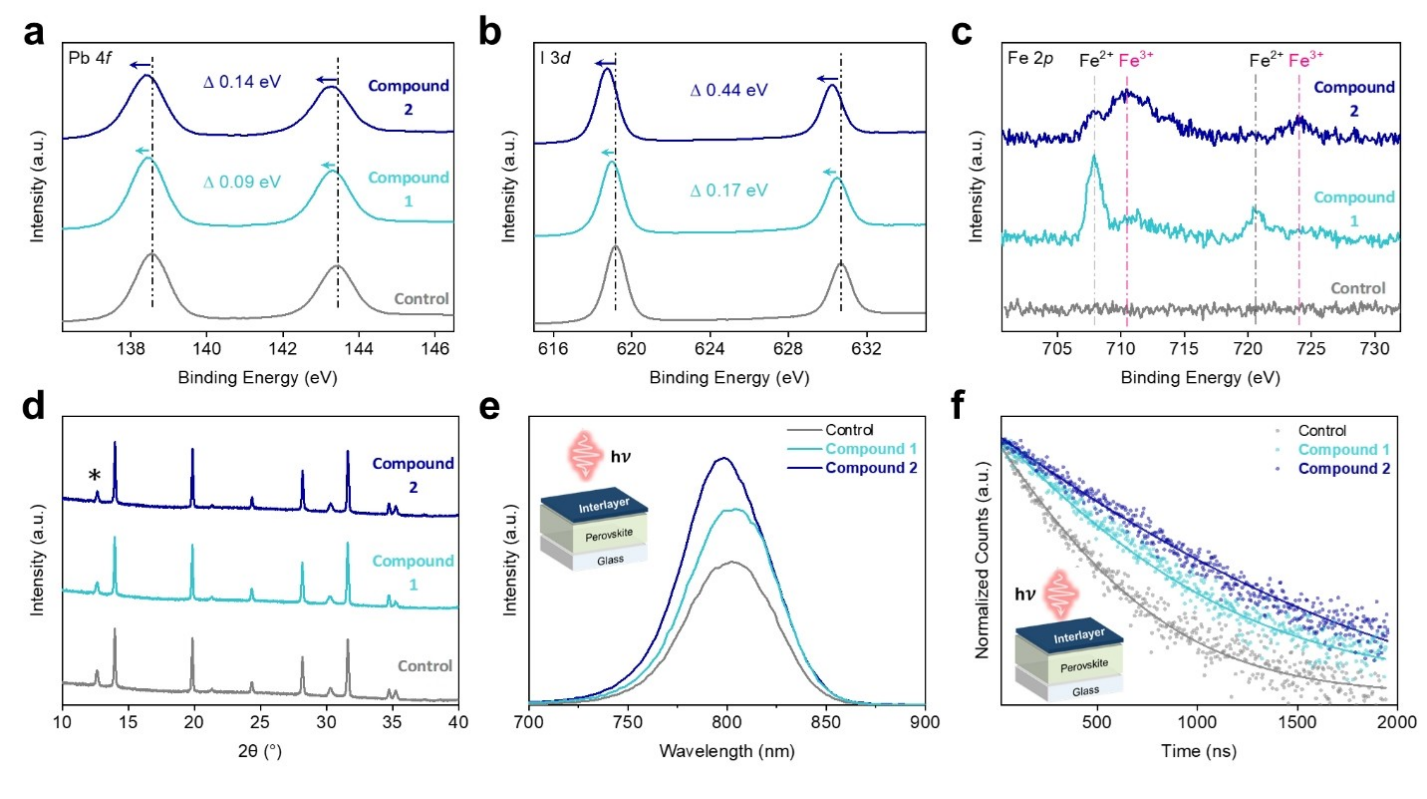
Surface chemistry. (a) and (b) Pb 4f and I 3d XPS characterization of control, **1** and **2** treated perovskite films. (c) Fe 2p XPS characterization of control and drop‐cast **1** and **2** treated PbI_2_ films. (d) XRD spectra of control, **1** and **2** treated perovskite films. * denotes the PbI_2_ peak at 12.7°. (e) and (f) Steady‐state PL and TRPL spectra of perovskite samples treated with different Fc‐based compounds, light incident from the perovskite film side. Insert shows the sample architecture used for luminescence characterization.

To support the molecular‐level insights provided by the DFT calculations, interactions of the Fc‐derived compounds with the perovskite surface were probed by X‐ray photoelectron spectroscopy (XPS; Figure 2a and b). Both samples showed obvious shifts in Pb 4f and I 3d peaks to lower binding energies, with **2** having the most significant effect, indicating strong surface binding.^[41]^ The presence of Fe on the surface was also confirmed (Figure S18). However, accurate peak analysis could not be performed due to low peak intensity and overlap with Cs^+^ signals (3d^5/2^ approx. 725 eV).^[42]^ To improve signal quality and better understand any potentially occurring redox interactions, we performed XPS on drop‐cast samples of **1** and **2** on PbI_2_ thin films (Figure 2c). Splitting of the Fe 2p peaks consistent with the presence of Fe^2+^ and Fe^3+^ signals at approximately 707, 721 eV, and 711, 724 eV, respectively, is observed in both spectra, indicating partial oxidation of the Fc moiety.^[43]^ Fitting of the Fe 2p^3/2^ peaks of compound **1** and **2** treated samples (Figure S19) revealed Fe^3+^/Fe^2+^ ratios of approximately 0.5 and 0.9, respectively, in accordance with the relative *E*_HOMO_ energies. To better understand the chemical interactions occurring at the surface, we analyzed residual Pb^0^ peaks in the Pb 4 f XPS spectrum (Figure S20), observing a lack of residual metallic lead in Fc‐treated samples. Moreover, X‐ray diffraction (XRD) measurements on perovskite (Figure 2d) and PbI_2_ thin films coated with the Fc‐derived compounds show a consistent decrease in PbI_2_ peak intensity at 12.7° (Figures S21 and S22). These observations are consistent with previous literature reports.[^26^, ^31^, ^44^] Ultraviolet‐visible absorption spectroscopy (UV/Vis), atomic force microscopy (AFM), and scanning electron microscopy (SEM) were also performed on treated perovskite layers (Figures S23–S25), and no obvious changes in morphology or light‐harvesting properties were noted. Therefore, we provide two possible explanations for the presence of Fe^3+^ surface states consistent with the above‐mentioned observations: (i) oxidation by I_2_ species[^45^, ^46^] generated from excess PbI_2_ in the perovskite composition and subsequent recovery of Pb^0^ traps^[44]^ and/or (ii) charge transfer from the Fc unit to the perovskite analogous to the observations made with cobaltocene.^[32]^

Steady‐state photoluminescence (PL), time‐resolved photoluminescence (TRPL) and space charge‐limited current (SCLC) measurements were performed to evaluate the ability of Fc‐derived compounds to passivate perovskite defects. The increase in PL intensity (Figure 2e) and lengthened average carrier lifetime (Figure 2f), from 1046 ns in the control sample to 1175 and 1525 ns in **1** and **2** samples, respectively (Table S5), indicate a suppression of non‐radiative surface recombination.^[47]^ SCLC measurements on electron‐only (Figure S26) devices with an ITO/SnO_2_/Perovskite/Fc‐interlayer/C_60_/BCP/Ag architecture revealed a consistent decrease in the trap‐filling voltage (VTFL) from 0.55 V in control samples to 0.36 V and 0.22 V in samples passivated with **1** and **2**, respectively, corresponding to over a two‐fold reduction in defect state density.^[48]^ Overall, these results show that **1** and **2** interact strongly with the perovskite surface, passivating surface defects and suppressing non‐radiative recombination, which are key factors affecting device *V*_oc_.

**Figure 3:**
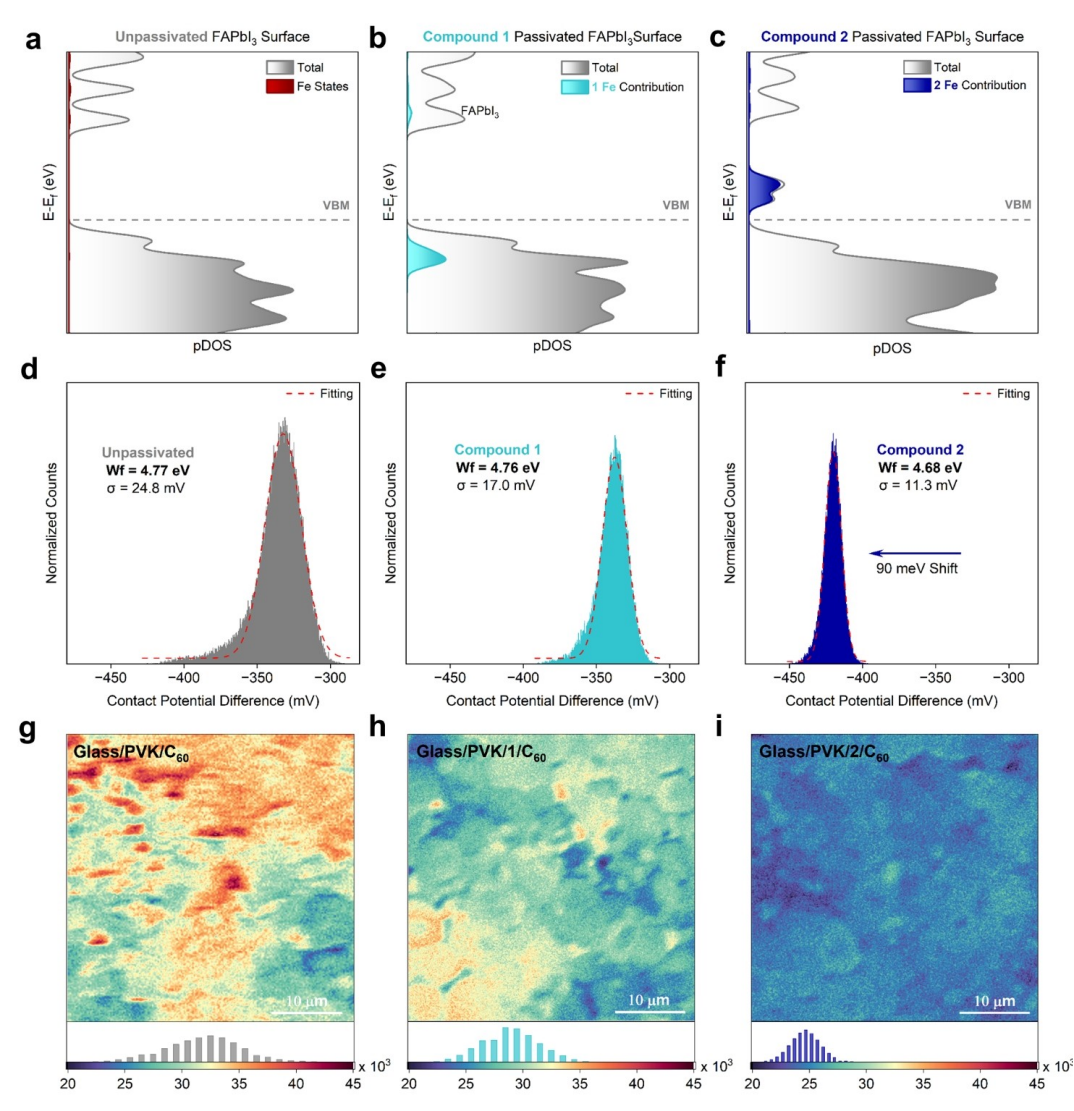
Role of *E*_HOMO_ in Controlling Interfacial Energetics. (a)–(c) Calculated pDOS plots of the perovskite surface functionalized with **1** and **2** with the highlighted contribution from Fe d orbitals showing their position relative to *E*_VBM_. (d)–(f) CPD distributions extracted from KPFM mapping data along with the calculated WF for perovskite films functionalized with the Fc‐based compounds. (g)–(i) PL mapping and respective PL intensity distribution histograms measured using glass/perovskite/Fc‐interlayer/C_60_ samples.

Having characterized the relevant chemical surface interactions, we explored the relationship between the Fc‐*E*_HOMO_ level and the electronic properties of the perovskite layer. DFT was adopted to calculate the projected density of states (pDOS) of the unpassivated and compound **1** and compound **2** passivated perovskite surface (Figures 3a–c). With no contribution from Pb‐ and I‐d orbitals expected in the valence band region of FAPbI_3_, all d‐state levels (dxy plotted) are attributed to Fe 3d *E*_HOMO_ levels. The relative positions of frontier Fe states in **1** and **2** below and above the perovskite *E*_HOMO_, respectively, are in excellent agreement with predictions made from CV and UPS (Figure 1b). Interestingly, no significant changes in valence band position or structure are observed, indicating negligible frontier orbital hybridization has occurred, and confirming the offset between *E*_HOMO_ and *E*_VBM_ is sufficient in both compounds. This was supported by calculations of the spatial charge distribution of the valence and conduction bands of passivated surfaces (Figures S27 and S28), where minimal electronic coupling between Fc states and the perovskite is noted.

Kelvin probe force microscopy (KPFM) measurements were performed to correlate the Fc‐*E*_HOMO_ energies with perovskite surface WF changes (Figure 3d–f and Figure S29). The unpassivated perovskite surface WF was measured at 4.77 eV with a standard deviation in contact potential difference (WF homogeneity) of 24.8 mV. Upon passivation with **1**, negligible changes in WF properties were measured, indicating that less readily oxidized Fc‐compounds (*E*_HOMO_<*E*_VBM_) are unable to affect surface potential, breaking the trend set out in Figure S1. On the other hand, the sample passivated with **2**, the more easily oxidized Fc‐center (*E*_HOMO_>*E*_VBM_), showed a significant decrease in WF to 4.68 eV, along with a homogenized potential distribution of 11.3 mV. This is consistent with reported literature values (60–160 meV) for Fc‐compounds with similar *E*_HOMO_ values. These results are also consistent with the decrease in perovskite WF reported for cobaltocene and benzyl viologen molecules attributed to surface charge transfer.^[32]^ Decreasing perovskite surface WF and homogenizing surface potential at the perovskite/ETL interface in p‐i‐n architectures are correlated with improved charge extraction efficiency and reduced minority carrier concentration, leading to improved PSC performance.[^14^, ^49^]

To support the observations made by KPFM and gain greater insight into the surface energetics at the perovskite/Fc interface, UPS measurements were conducted (Figures S30 and S31). Using the secondary electron cutoff region to determine the perovskite WF, values of 4.75, 4.76, and 4.67 eV were calculated for unpassivated, PVK/**1** and PVK/**2** samples, respectively, in excellent agreement with the KPFM results. A concomitant increase in the VBM of **1**‐ and **2**‐passivated samples of 0.25 and 0.03 eV was also observed (Figure S32). The slight increase in valence and conduction bands, along with a lack of WF change, provides further evidence that the less readily oxidized compound **1** cannot favorably tune interfacial energetics. The decreased WF and minor alteration of the *E*_VBM_ in the compound **2** sample on the other hand should allow for downward band bending at the perovskite surface (Figure S33), depleting minority charge carriers (holes) and boosting charge extraction to C_60_.^[19]^

To test the validity of the insights gained from KPFM and UPS experiments, we measured the efficiency and homogeneity of charge extraction at the PVK/C_60_ interface by performing PL mapping on Glass/PVK/Fc/C_60_ samples, as seen in Figures 3g–i. In the unpassivated sample, a broad distribution in the photoluminescence intensity is observed on contact with the ETL, consistent with inhomogeneous charge extraction. The introduction of **1** caused a slight decrease in the average PL intensity and mildly ameliorated the PL homogeneity, indicating a minor improvement in charge extraction potentially attributed to the decreased standard deviation in the WF (Figure 3e).[^20^, ^30^, ^47^] These results suggest that although less easily oxidized Fc compounds do not significantly impact the energetic properties of the perovskite surface, the delocalization of the Fc structure may still play a role in charge transfer to C_60_. Following the deposition of **2**, however, a substantial decrease in the PL intensity was observed, indicating a significant increase in charge extraction with much‐improved homogeneity. This is in excellent agreement with KPFM and UPS results. We have therefore elucidated the direct correlation between the Fc‐*E*_HOMO_ level and the ability of Fc‐based compounds, having *E*_HOMO_>*E*_VBM_, to decrease and homogenize the perovskite surface WF. In turn, the improved interfacial energetics led to enhanced charge extraction to the ETL, thus effectively allowing chemists to synthetically control charge extraction by tuning the electrochemical potential of Fc‐based interlayers.

**Figure 4:**
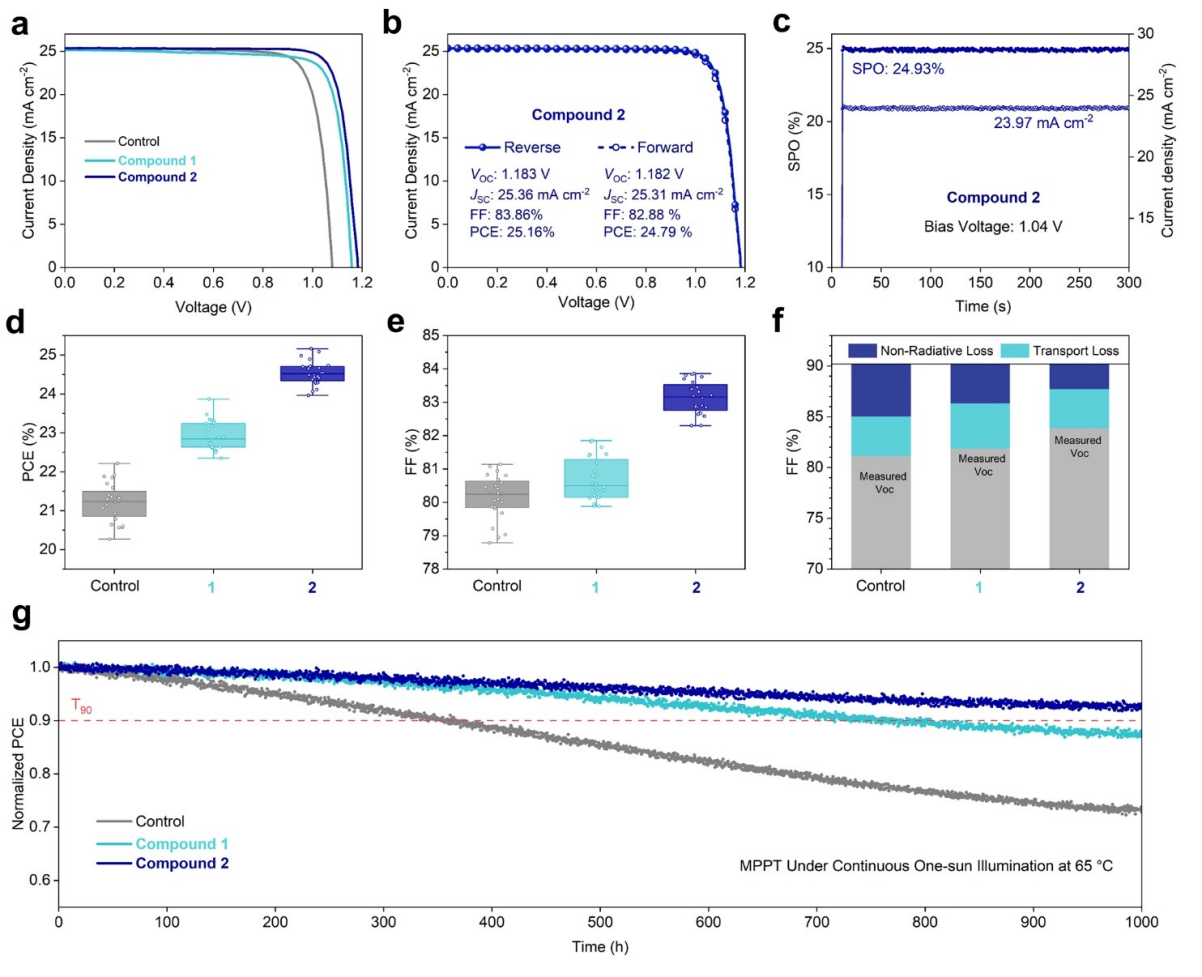
Photovoltaic Performance and Stability (a) *J–V* curves of best‐performing PSCs with and without the Fc‐based compounds. (b) Forward and reverse scan of the champion device treated with compound **2** interlayer. (c) SPO and corresponding stabilized *J*_sc_ of the champion two devices. (d) and (e) PCE and FF distribution extracted from *J–V* curves of 24 individual devices. (f) FF loss analysis revealing the dominant loss pathways in control, **1**, and **2** functionalized PSCs. (g) Normalized PCE evolution of encapsulated devices measured at MPP under continuous operation at one‐sun intensity at 65 °C in N_2_.

The photovoltaic performance of PSCs featuring interlayers of **1** and **2** on a device architecture consisting of ITO/NiOx/SAM/Perovskite/Fc‐Interlayer/C_60_/BCP/Ag (Figure S34) was evaluated using current density‐voltage (*J–V*) curves (Figure 4a). The champion control device achieved a PCE of 22.21 % with a short‐circuit current density (*J*_sc_) of 25.34 mA cm^−2^, a *V*_oc_ of 1.080 V and a FF of 81.14 %, as shown in Figure S35. A modest improvement in PCE to 23.87 % (*J*_sc_=25.16, *V*_oc_=1.159, FF=81.85 %) was observed after the introduction of **1**, whereas a significant boost in efficiency to 25.16 % (*J*_sc_=25.36, *V*_oc_=1.183, FF=83.86 %) was attained with **2** (Figure 4b and Table S6). The stabilized power output (SPO) and photocurrent of the best‐performing device functionalized with **2** were measured to be 24.93 % and 23.97 mA cm^−2^, respectively, after holing at the maximum power point (MPP) for 300 seconds (Figure 4c and Figure S36). To ensure the validity of our solar cell analysis, we measured the external quantum efficiency (EQE) spectra of champion devices, where integrated *J*_sc_ values matched well with *J–V* measurements (Figure S37).^[50]^ Statistical analysis of 24 individual devices revealed that improved PCEs in compound **1** samples resulted primarily from increased *V*_oc_, whilst samples with **2** benefitted from significant enhancements to both *V*_oc_ and FF, as shown in Figures 4d, e and Figure S38. *V*_oc_ and FF losses in PSCs are primarily controlled by non‐radiative recombination originating from bulk and surface traps as well as energetic misalignment at the perovskite/ETL interface.^[51]^ Therefore, the trend in measured *V*_oc_ is consistent with the respective ability of compounds **1** and **2** to passivate defects, reducing non‐radiative surface recombination. Average FF (Figure 4e) values on the other hand followed the relative shifts in surface WF, where little difference was observed between control (80.15 %) and compound **1** (80.67 %) functionalized devices, whilst a significant improvement was noted following the deposition of **2** (83.13 %), suggesting improved charge extraction.[^19^, ^52^]

To better understand the origin of the FF losses (Figure 4f) we performed intensity‐depended *V*_oc_ measurements and calculated ideality factors of 1.38, 1.41 and 1.25 for control, **1** and **2** samples (Figure S39), respectively, according to **Note** **S3**. While both **1**‐ and **2**‐functionalized devices showed decreased non‐radiative losses of 3.89 % and 2.50 %, respectively, compared to 5.16 % for control devices, only **1** showed an increased transport loss of 4.46 % compared to 3.9 % for control and 3.84 % for compound **2**. This observation is directly attributable to the successful surface passivation enabled by both compounds and the improved interfacial energetics attained only with compound **2**. As such, we have successfully separated the passivating and energetic contributions of Fc‐interlayers in inverted PSCs by correlating the chemical and electrochemical properties of the molecules involved to device *V*_oc_ and FF.

Additionally, the effects of the Fc‐based interlayers on the long‐term operational stability of PSCs were studied by performing MPP tracking on encapsulated devices held at 65 °C in a N_2_ atmosphere (Figure 4g).^[53]^ Devices treated with compounds **1** and **2** showed impressive stabilities, retaining >87 % and >92 % of their initial efficiency after 1000 hours of testing, respectively. In contrast, the control device efficiency fell to approximately 73 % of its initial value. This significant improvement in stability can be correlated to the reduction in residual PbI_2_, known to increase the rate of PSCs degradation, and to the relative ability of the Fc‐based molecules to be oxidized by I_2_ and thus block the migration of the degradation product (Figure S19).^[54]^

Lastly, we verified the conclusions drawn herein by performing KPFM and *J–V* measurements using the previously reported FcTc_2_ compound (Figure S40 and Figure S41). The *E*_HOMO_ energy of FcTc_2_ (−5.23 eV^[30]^) is practically identical to that of compound **2**, therefore a similar shift in the perovskite surface WF is expected. Indeed, a WF of 4.68 eV was measured for the FcTc_2_‐treated perovskite layer (Figure S40). *J–V* measurements (Figure S41) provide further support for our hypothesis as the champion FcTc_2_ treated device could achieve a PCE of 24.67 % with an excellent FF of 83.04 %, significantly higher than compound **1** and approaching **2**. Likewise, the measured *V*_oc_ of 1.178 V is significantly higher than compound **1** but lower than compound **2**. This can be attributed to the enhanced passivation enabled by the furyl substituents in **2** as opposed to the thiophene substituents present in FcTc_2_, consistent with the DFT results presented above. Critically, the structure‐function‐efficiency relationships outlined herein consistently explain previously reported observations involving Fc‐based interlayers applied in PSCs (Figure S1).[^12^, ^26^, ^30^] Therefore, the concept of perovskite surface WF tuneability through organometallic *E*_HOMO_ energies, and passivation of undercoordinated Pb^2+^ atoms by coordinating side groups on the Fc framework, should inspire novel highly tailored organometallic interlayers achieving exceptional PSC performance.

## Conclusion

We have shown that the synthetic flexibility of the Fc framework can be leveraged to rationally tune the passivating and energetic properties of organometallic interlayers simultaneously using two structurally similar molecules, **1** and **2**, with differing oxidation potentials. Both compounds exhibited strong interactions with undercoordinated Pb^2+^ centers through the appended lone pairs on the carboxyl and furyl moieties, leading to a reduction in non‐radiative surface recombination. Measuring the change in perovskite WF following the deposition of the Fc‐based molecules revealed that only the more easily oxidized **2** could decrease and homogenize the surface WF, whilst the less readily oxidized compound **1** had a negligible impact. These observations are consistent with the improvements in the inverted PSC device parameters where compound **1** improved primarily the *V*_oc_ whilst **2** boosted both the *V*_oc_ and FF, ultimately affording inverted PSCs with PCEs reaching 25.16 % that maintain >92 % of their initial efficiency after 1,000 hours of accelerated MMP aging. By correlating the electrochemical potential of Fc‐derived molecules to the electronic properties of the perovskite layer and the resulting impact on charge extraction, we have highlighted a new avenue of tuneability for Fc‐based interlayers.

## Supporting Information

Further details on experimental procedures, synthetic characterization including NMR, CV, UV/Vis, single‐crystal XRD, DFT calculation details, XPS, SEM, AFM, KPFM, SCLC, EQE and *J–V* curves are presented in the Supporting Information document. The authors have cited additional references within the Supporting Information.

## Accession Codes

CCDC 2390047 and 2390048 contain the supplementary crystallographic data for Compounds **1** and **2** respectively. These data can be obtained free of charge via www.ccdc.cam.ac.uk/data_request/cif, or by emailing data_request@ccdc.cam.ac.uk, or by contacting The Cambridge Crystallographic Data Centre, 12 Union Road, Cambridge CB2 1EZ, UK; fax: +44 1223 336033.

## Conflict of Interests

The authors declare no conflict of interest.

1

## Supporting information

As a service to our authors and readers, this journal provides supporting information supplied by the authors. Such materials are peer reviewed and may be re‐organized for online delivery, but are not copy‐edited or typeset. Technical support issues arising from supporting information (other than missing files) should be addressed to the authors.

Supporting Information

## Data Availability

The data that support the findings of this study are available from the corresponding author upon reasonable request.
