# Supplementary material for: Modulating Perovskite Surface Energetics Through Tuneable Ferrocene Interlayers for High‐Performance Perovskite Solar Cells
Source: Angew Chem Int Ed Engl. 2025 Jan 26;64(14):e202424041. doi: 10.1002/anie.202424041 (PMC11966690; doi:10.1002/anie.202424041)
Supplement: Supplementary file 1 — Supporting Information [file ANIE-64-e202424041-s001.pdf]

Angewandte Chemie
Eine Zeitschrift der Gesellschaft Deutscher Chemiker

Supporting Information

Modulating Perovskite Surface Energetics Through Tuneable Ferrocene Interlayers for High-Performance Perovskite Solar Cells

F. Vanin, W. D. J. Tremlett, D. Gao, Q. Liu, B. Li, S. Li, J. Gong, X. Wu, Z. Li, R. K. Brown, L. Qian, C. Zhang, X. Sun, X. Li, X. C. Zeng, Z. Zhu*, N. J. Long**

Supporting Information

Modulating Perovskite Surface Energetics Through Tuneable Ferrocene Interlayers for High-Performance Perovskite Solar Cells

Francesco Vanin,[a,b]‡ William D. J. Tremlett,[b]‡ Danpeng Gao,[a]‡ Qi Liu,[c]‡ Bo Li,[a] Shuai Li,[a] Jianqiu Gong,[a] Xin Wu,[a] Zhen Li,[a] Ryan K. Brown,[b] Liangchen Qian[a], Chunlei Zhang,[a] Xianglang Sun,[a] Xintong Li,[a] Xiao Cheng Zeng*[c], Zonglong Zhu*[b], and Nicholas J. Long*[a]

‡ These authors contributed equally

[a] F. Vanin, Dr. D. Gao, Dr. B. Li, S. Li, J. Gong, Dr. X. Wu, Dr. Z. Li, L. Quian, C. Zhang, X. Sun, X. Li, Prof. Z. Zhu
Department of Chemistry
City University of Hong Kong
Kowloon 999077, Hong Kong
E-mail: zonglzhu@cityu.edu.hk

[b] Dr. W. D. J. Tremlett, Dr. R. K. Brown, Prof. N. L. Long
Department of Chemistry
Imperial College London
MSRH Building, White City Campus, W12 0BZ, London, UK
E-mail: n.long@imperial.ac.uk

[c] Dr. Q. Liu, Prof. X. C. Zeng
Department of Materials Science & Engineering
City University of Hong Kong
Kowloon 999077, Hong Kong
E-mail: xzeng26@cityu.edu.hk

Methods

Materials

Formamidinium iodide (FAI), methylammonium bromide (MABr), and cesium iodide (CsI) and 1,8-octanediamine dihydroiodide (ODADI) were purchased from Dysol (Australia). Lead iodide (PbI2) and lead bromide (PbBr2), (2-(3,6-Dimethoxy-9H-carbazol-9-yl)ethyl)phosphonic acid (MeO-2PACz) and (4-(3,6-Dimethyl-9H-carbazol-9-yl)butyl)phosphonic acid (Me-4PACz) were purchased from TCI (Japan). C60, methylammonium chloride (MACl) and bathocuproine (BCP, 99.9%) were purchased from Xi'an Polymer Light Technology Corporation (China). Nickel oxide nanoparticles (NiO_x, 7 nm average particle size) was purchased from advanced Election Technology Co., Ltd (China) High purity silver was purchased from commercial sources. Aluminum chloride (99%), 2-furoyl chloride (95%) and Ferrocene (98%, purified by sublimation prior to use) were purchased from Sigma-Aldrich. Copper(I) oxide (97%) was purchased from Acros Organics. Furoic acid (>98%) was purchased from Tokyo Chemical Industry Ltd. Diiodoferrocene (FcI_2)[1], and ferrocenyl-bis-furyl-2-ketone (**1**)[2] were prepared using modified literature procedures. Glass substrates patterned with indium tin oxide (ITO) (15 Ω sq-1) were received from Mishi Tech. Co., Ltd. (China). Dimethylformamide (DMF), dimethyl sulfoxide (DMSO) and chlorobenzene (CB) were purchased from J&K (China) and used as received. Dichloromethane (CH_2Cl_2) and *n*-hexane were purchased from VWR Chemicals, and deuterated chloroform ($CDCl_3$) was purchased from Sigma-Aldrich. Anhydrous acetonitrile (CH_3CN) and dichloromethane (CH_2Cl_2) were dried through a solvent purification system under a nitrogen atmosphere (Inert PureSolv) and transferred into Schlenk flasks that were dried under vacuum and purged with N_2 prior to use.

Synthesis of Ferrocenyl-bis-furan-2-ketone (**Compound 1**)

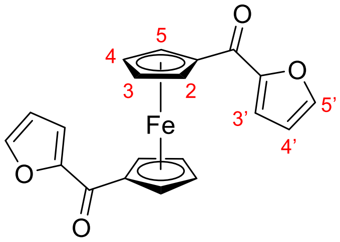

Ferrocene (3.02 g, 16.2 mmol) was added to a stirred solution of $AlCl_3$ (10.8 g, 81.1 mmol) and 2-furoyl chloride (8.0 mL, 81.1 mmol) in anhydrous CH_2Cl_2 (120 mL) and stirred rt for 18 h. The reaction mixture was cooled to 0 °C and ice-cooled water (200 mL) added. The mixture was separated, and the aqueous phase extracted with CH_2Cl_2 (100 mL × 3). The combined organic layers were washed with satd. aq. $NaHCO_3$ (200 mL), water (200 mL) and brine (200 mL), dried over Na_2SO_4, filtered and concentrated *in vacuo*. The crude residue was purified by flash chromatography (*n*-hexane/EtOAc 4:1 → 1:1) and the resulting product recrystallized from CH_2Cl_2 and *n*-hexane to afford **1** (989 mg, 16%) as dark red crystals. R_f 0.37 (*n*-hexane/EtOAc 1:1); ^{1}H NMR (400 MHz, $CDCl_3$): δ 7.56 (dd, 2H, $^3J_{HH}$ = 1.7 Hz, $^4J_{HH}$ = 0.9 Hz, 2 × H-5'), 7.28 (dd, 2H, $^3J_{HH}$ = 3.7 Hz, $^4J_{HH}$ = 0.9 Hz, 2 × H-3'), 6.54 (dd, 2H, $^3J_{HH}$ = 3.5 Hz, $^3J_{HH}$ = 1.7 Hz, 2 × H-4'), 5.17 (pseudo-t, 4H, J = 2.0 Hz, 2 × H-3 and H-4), 4.57 (pseudo-t, 4H, J = 2.0 Hz, 2 × H-2 and H-5); ^{13}C{^{1}H} NMR (100 MHz, $CDCl_3$): δ 183.8 (2 × C, 2 × C=O), 153.6 (2 × C, 2 × C-2'), 145.8 (2 × CH, 2 × C-5'), 117.3 (2 × CH, 2 × C-3'), 112.3 (2 × CH, 2 × C-4'), 79.3 (2 × C, 2 × C-1), 74.4 (4 × CH, 2 × C-2 and C-5), 72.6 (4 × CH, 2 × C-3 and C-4); MS (ESI$^+$): *m/z* 375.0320 $[M + H]^+$ (m_{calc} 375.0320); Calcd for $C_{20}H_{14}FeO_4$: C 64.20, H 3.77. Found: C 64.27, H 3.50.

Synthesis of Ferrocenyl-bis-furan-2-carboxylate (**Compound 2**)

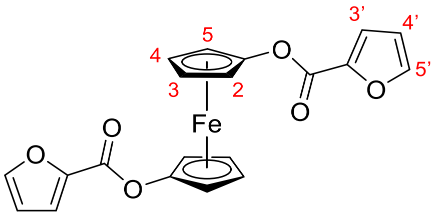

FcI_2 (311 mg, 0.71 mmol), Cu_2O (152 mg, 1.07 mmol) and furoic acid (239 mg, 2.13 mmol) was refluxed for 24 h in anhydrous CH_3CN (30 mL). The rection mixture was diluted with CH_2Cl_2 (30 mL), filtered and the filtrate washed with satd aq $NaHCO_3$ (30 mL × 3). The organic

phase was dried over Na_2SO_4, filtered and concentrated *in vacuo*. The crude residue was purified by flash chromatography (CH_2Cl_2/*n*-hexane 1:1 → CH_2Cl_2 neat) and recrystallised from CH_2Cl_2/*n*-hexane to afford orange-yellow crystals of **2** (205 mg, 71%). R_f 0.43 (CH_2Cl_2/*n*-hexane 4:1); ^{1}H NMR (400 MHz, $CDCl_3$): δ 7.55 (d, 2H, $^3J_{HH}$ = 1.8 Hz, 2 × H-5'), 7.19 (d, 2H, $^3J_{HH}$ = 3.5 Hz, 2 × H-3') 6.49 (dd, 2H, $^3J_{HH}$ = 3.6 Hz, $^3J_{HH}$ = 1.8 Hz, 2 × H-4'), 4.69 (pseudo-t, 4H, *J* = 2.1 Hz, 2 × H-3 and H-4), 4.09 (pseudo-t, 4H, *J* = 2.1 Hz, 2 × H-2 and H-5); ^{13}C{^{1}H} NMR (100 MHz, CDCl3): δ 156.6 (2 × C, 2 × C=O), 146.9 (2 × CH, 2 × C-5'), 144.2 (2 × C, 2 × C-2'), 118.9 (2 × CH, 2 × C-3'), 116.3 (2 × C, 2 × C-1), 112.1 (2 × CH, 2 × C-4'), 64.8 (4 × CH, 2 × C-2 and C-5), 62.2 (4 × CH, 2 × C-3 and C-4); MS (ESI$^+$): *m/z* 406.0144 $[M]^+$ (m_{calc} 406.0140); Calcd for $C_{20}H_{14}FeO_6$: C 59.14, H 3.47. Found: C 59.40, H 3.47.

Perovskite Solar Cell Fabrication

Glass/ITO (15 Ω sq^{-1}) substrates were cleaned by sequential sonication in detergent, deionized water and ethanol for 20 minutes respectively before being stored in a 60 °C drying oven. The dry glass/ITO substrates were then treated with oxygen plasma for 15 minutes and allowed to cool prior to use.

The NiO_x ink was prepared by dispersing NiO_x nanoparticles (average size of 7 nm) in deionized water at a concentration of 10 mg/ml and filtered using a 0.22 µm PTFE syringe filter. The as-prepared NiO_x ink was spin-coated onto the glass/ITO substrate at a speed of 4,000 r.p.m. for 30s and annealed at 150 °C for 30 min in ambient air before being transferred to a N_2-filled glovebox. A mixed self-assembled monolayer (SAM) solution was prepared by combining 0.3 mg/ml solutions of MeO-2PACz and Me-4PACz in ethanol in a 1:1 v/v ratio. The mixed SAM solution was spin-coated onto the glass/ITO/NiO_x substrate at 4,000 r.p.m. for 30s and annealed at 100 °C for 10 min. Subsequently, the SAM layer was washed by spin-coating ethanol at 4,000 r.p.m. for 30s and annealed at 100 °C for 5 min.

The $(FA_{0.98}MA_{0.02})_{0.95}Cs_{0.05}Pb(I_{0.98}Br_{0.02})_3$ perovskite precursor solution (1.55 M) was prepared by dissolving CsI, FAI, MABr, PbI_2 and $PbBr_2$ in 1 ml mixed DMF:DMSO (4:1 v/v) with the addition of 12.6 mol% MACl and 10 mol% excess of PbI_2 in a N_2-filled glovebox. Prior to device fabrication 0.04 mol% of ODADI was added to the precursor solution and allowed to

stir for 2 h. Deposition of the perovskite layer was performed by spin-coating 80 µl the as-prepared precursor on the glass/ITO/NiO_x/SAM substrate at 1,000 r.p.m. for 10 s and subsequently 5000 r.p.m. for 40 s, 350 µl of CB was dripped onto the center of the film 10 s before the end of the spin-coating procedure. The substrates were then directly annealed at 100 °C for 30 min. All procedures were conducted in a N_2-filled glovebox with a controlled temperature between 18-23 °C and water and oxygen levels controlled <5 ppm.

For **1**- and **2**-based devices, the respective compound was fully dissolved in CB at an optimized concentration of 1 mg/ml at room temperature in a N_2-filled glovebox. Subsequently, 100 µ of the as-prepared solution was dynamically spin-coated on the perovskite layer at 5,000 r.p.m for 30 s and then annealed at 100 °C for 5 min.

Finally, 25 nm of C60 at a rate of 0.5 Å s^{-1} followed by 6 nm BCP at a rate of 0.2 Å s^{-1} and a 100 nm silver electrode at a rate of 1.0 Å s^{-1} were thermally evaporated, respectively, under high vacuum (< 4 × 10−6 Torr).

Stability Tests

Device encapsulation was performed by evenly applying a high-hardness encapsulation adhesive (BONLE, model 9213) around the perimeter of glass encapsulation covers with suitable dimensions and a thickness of approximately 0.7 mm. The adhesive-coated encapsulation cover was adhered to the surface of the perovskite solar cell and cured by illuminating a 30W UV lamp operating at 365 nm r for 30. The edges of the encapsulation cover were sealed in contact with the device using polyisobutylene encapsulation tape.

Long-term operational stability tests were performed on encapsulated perovskite solar cells illuminated by a 1 sun equivalent LED lamp in a N_2-filled glovebox (maintaining water and oxygen levels <10 ppm) at 65 °C. The perovskite solar cells were biased at a maximum-power-point (MPP) voltage and the power output was tracked using a multi-potentiostat (CHI1040C, CH Instruments, Inc.). During MPP testing current density-voltage curves of the devices were

obtained every 12 h to adjust the load. A thermostatic control system was applied to keep the device at 65 °C.

Density functional theory (DFT) calculations

The *first principles* DFT calculations were performed with the Vienna Ab Initio Simulation Package (VASP 6.4)[3–5] to study the geometric and electronic structures of all the bare and Fc-treated $FAPbI_3$ perovskite surface structures with (001) PbI_2 terminal. Unless otherwise specified, the generalized gradient approximation exchange-correlation functional of Perdew-Burke-Ernzerhof (PBE)[5] was adopted in the DFT calculations. The electronic constituents are 2s 2p for O C and N, 3d 4s for Fe, 6s 5d for Pb and 1s for H. For all the bare and Fc-treated perovskite surface structures, we adopted 2 × 2 × 1 k-point mesh for 2D vacuum surfaces, generated by the Monkhorst-Pack scheme, for detailed properties obtained with PBE functional. Considering the strong relativistic effect of the heavy element atoms like Pb, the spin-orbit coupling (SOC) effect was taken into account for all the electronic property calculations except geometric configuration optimization. For geometric optimization of the isolated Fc compounds in large vacuum boxes, we adopted the Γ-only k-point mesh. The projector augmented wave (PAW) pseudopotentials with the cut-off energy of 600 eV were employed. Considering the van der Waals interaction between the hydrogen atoms and high-electronegativity groups, the PBE with the DFT-D3 dispersion correction of Grimme with zero-damping[6–8] was applied to optimize the geometric structures. During the optimization of the geometries, all structures were allowed to relax to ensure that each atom was in mechanical equilibrium without any residual force larger than 10^{-4} eV/Å. A 25-Å vacuum layer was adopted on the surface structures to prevent the interaction between the fixed terminal layers. For surface binding energy calculation, we adopted the definition of $\varepsilon = \frac{E_{slab} - (\Sigma_i^n E_{i(comp)})}{2S}$, in which ε represents the surface binding energy value, E_{slab} is the optimized energy of the surface structure while $E_{i(comp)}$ is the optimized energy of every component of the heterostructure in independent vacuum boxes, the S term on the denominator represents the surface area of the structure. In the system considered here, S is identical as all the structures are identical.

Characterization

Nuclear magnetic resonance (NMR) spectra were recorded at ambient temperature on a Bruker Avance 400 MHz spectrometer using 5 mm 507-pp NMR tubes and processed using MestReNova v14.3.2. NMR spectra were calibrated to the residual solvent peaks of $CDCl_3$ at 7.26 and 77.2 ppm for ^1H and $^{13}C\{^1H\}$, respectively. Peak assignment was aided through the use of COSY, edited HSQC and HMBC experiments.

High-resolution mass spectrometry data were recorded by direct injection on a Waters LCT time-of-flight (ToF) Mass Spectrometer using electrospray ionization (ESI) conducted by Malgorzata Puchnarewicz of the Mass Spectrometry Service at Imperial College London.

Elemental analyses were analyzed using a ThermoFlash 2000 Analyzer by the Elemental Analysis Service at London Metropolitan University.

Single crystal X-ray diffraction measurements were performed using an Agilent Xcalibur PX Ultra A diffractometer and collected at 173 K. The structures were solved and refined using Olex2,[9] SHELXTL[10] and SHELX-2013.[11]

Cyclic voltammetry (CV) analyses were conducted on a Gamry 600TM potentiostat to record the redox potentials associated with each ferrocene compound. The samples were analyzed in a cell comprising of glassy carbon working, platinum counter and silver pseudo reference electrode. The samples (2 mM) were dissolved in an electrolyte solution of tetrabutylammonium hexafluorophosphate (0.1 M) in anhydrous and degassed CH_2Cl_2, and analyzed at scan rates of either 20, 50, 100, 250, 500 or 1000 mVs^{-1}. Each sample was referenced to a ferrocene/ferrocenium (Fc/Fc^+) internal reference and all scans were corrected for solution resistance with the values obtained from potentiostatic electrochemical impedance spectroscopy (EIS) measurements.

Ultraviolet-visible (UV-Vis) absorbance spectra of samples in solution were obtained with an Agilent Technologies Cary 60 UV-Vis Spectrophotometer. All samples (5–1000 μM) were analyzed at room temperature using a quartz cell with a path length of 1 cm in CH_2Cl_2. UV-Vis. absorbance spectra of thin film samples deposited on quartz glass substrates were conducted on a PerkinElmer Lambda 1050 UV/Vis/NIR Spectrophotometer.

Photoluminescence (PL) and time-resolved photoluminescence (TRPL) spectra were collected using an Edinburgh FLS1000 spectrophotometer with an excitation wavelength of 485 nm.

X-ray photoelectron spectroscopy (XPS) measurements were conducted on a ThermoFisher Scientific K-Alpha$^+$ instrument at pressures $<10x10^{-9}$ mbar using an AlK_α X-ray source with an energy of 1,486.6 eV. All reported spectra were charge-corrected by setting the adventitious carbon 1s energy to 285.0 eV. Spectral fitting was performed using the CasaXPS software.[12]

Ultraviolet photoelectron spectroscopy (UPS) was conducted in a surface analysis system (VG ESCALAB 220i XL) equipped with a He discharge lamp (hv = 21.22 eV).

X-ray diffraction (XRD) data were collected in the reflection mode at room temperature on a Philips X'Pert diffractometer equipped with a CPS 180 detector using monochromated Cu-Kα (λ = 1.5418 A) radiation.

All AFM-based characterization (AFM and KPFM) was conducted through Bruker Dimension ICON, and Ti/Ir coated silicon tips (ASYELELC-01-R2) with a resonance frequency at ~ 58-97 KHz were used in Scanning Kelvin Probe Microscopy (SKPM) with the work function internally calibrated to an Au. All AFM-based experiments were performed in air.

Scanning electron microscopy (SEM) images were acquired using a Thermo Fisher Scientific QUATTROS SEM.

The photovoltaic performance characteristics (J-V curves) of perovskite solar cells were conducted in a N2-filled glovebox at room temperature using a Xenon lamp solar simulator (Enlitech, SS-F5, Taiwan). The light power was calibrated to 100 mW cm-2 by a silicon S7 reference cell (with a KG2 filter). All the devices were measured using a Keithley 2400 source meter under a sweep mode of reverse scan (from 1.20 V to -0.01 V) and forward scan (from -0.01 V to 1.20 V) with the scan rate of 0.01 V s-1, and the delay time was 10 ms. No pre-condition was needed before measurement. The active area was defined and characterized as 0.0419 cm2 by metal shadow mask.

The stabilized power output was conducted by monitoring the stabilized current density output at the MPP bias (extracted from the reverse scan J-V curves). External quantum efficiency (EQE) measurements were carried out using a QE-R EQE system (Enlitech, Taiwan).

Supplementary Note 1: HOMO Level Calculations from Cyclic Voltammetry Data

Fc is an iconic molecule in electrochemistry, displaying reversible, stable and fast one-electron oxidation to ferrocenium (Fc^+), and is widely employed as an internal reference in CV experiments to determine the highest occupied molecular orbital (HOMO) level of organic semiconductors.[13–15] To determine the absolute HOMO energy of Fc compounds the midpoint of the oxidation and reduction peak potentials, known as the half-wave potential ($E_{1/2}$), is measured against an internal Fc/Fc^+ reference. This is used to approximate the formal potential ($E^{0\prime}$) of the corresponding redox couple referenced to the normal hydrogen electrode (NHE).[15–17] Conversion of $E_{1/2}$ values from the electrochemical scale (in mV vs Fc/Fc^+) to the vacuum scale (in eV) can then be performed using Equation (1), where the conversion parameter of 5.1 eV corresponds to the redox potential of Fc in the vacuum scale (see below). The reversibility and stability of the redox events in **1** and **2** were ensured by plotting the peak current versus the square root of the scan rate and peak position versus scan rate (**Figs. S7-S10**).

$$E_{HOMO}(eV) = -\left(E_{1/2_{vs.Fc/Fc^+}} + 5.1\right)(eV) \quad (1)$$

The appropriate factor for converting measured electrochemical potentials to the vacuum scale using Fc/Fc^+ as a reference couple is still debated due to the various reported values for the conversion of Fc/Fc^+ potentials to the NHE and subsequently the relation of the NHE to the vacuum scale.[18,19] The conversion of 5.1 eV used herein was calculated according to previous reports[15,18] using a value of 4.44 eV (Hansen and Hansen)[19] as equivalent to 0 V vs. NHE, a value of 0.24 V (Bard and Faulkner)[20] as the potential of the standard calomel electrode (SCE) vs. NHE and a value of 0.41 V as the potential of Fc/Fc^+ vs. the SCE. Using this conversion factor, we observed excellent agreement between the alignment of the **1** and **2** HOMO levels relative to the measured perovskite valance band maximum and the calculated projected density of states obtained through density functional theory.

Note: all HOMO energies quoted for other Fc compounds in this report have been calculated using Equation (1) and values for the redox potentials of other Fc species were taken from literature reports and converted accordingly.

Supplementary Note 2: Details on Single Crystal X-ray Structures.

Crystal Structure of **1**:

The Fe atom in the structure of **1** was found to be disordered across symmetry-related positions in only 50% occupancy. The packing between cyclopentadienyl-furanone ligands is such that Cp centroid–centroid distances above and below the Cp ring are identical (3.3800(4) Å), allowing the ferrocene to be equally defined using ligands 1 and 2, then 3 and 4 (and so on) or 2 and 3, then 4 and 5 (and so on) (see **Fig. S13**). This makes it appear as a continuous Fe–Cp–Fe–Cp polymer, but with 50% occupancy iron atoms so that it is instead stacked ferrocenes.

Crystal Structure of **2**:

The crystal of **2** that was studied was found to be a two-component merohedral twin in a *ca.* 94:06 ratio (BASF = 0.0569(11)) with the two lattices related by the twin law [-1 0 0 0 -1 0 1 0 1 2]. The structure contained two crystallographically independent molecules (**2**-A and **2**-B) in the asymmetric unit. The Fe1 atom in the molecule **2**–B was found to be disordered across two positions in a *ca*. 93:07% occupancy respectively in a similar fashion as described previously for **1**. The packing of the cyclopentadienyl-furanoate ligands are such that the Cp centroid–centroid separations "above" and "below" the ring are very similar (3.332(5) and 3.810(5) Å respectively) allowing the ferrocene to be defined by either position. What appears as a continuous polymer is in fact stacked ferrocenes. The Fe center of the minor occupancy was refined isotropically (all other atoms were refined anisotropically). The absolute structure of **2** was determined by use of the Flack parameter [x^+ = –0.013(14)].

Supplementary Note 3: FF loss calculation.

The FF loss between the Shockley–Queisser limit and measured FF value is composed of nonradiative loss and resistance loss, and the maximum FF (FFmax) can be empirically calculated with the equation:

$$FF_{max} = \frac{\nu OC - \ln(\nu OC + 0.72)}{\nu OC + 1}$$

$$\nu OC = \frac{qV_{OC}}{nk_B T}$$

Where denotes elementary charge, V_{OC} is the measured open-circuit voltage, n is the ideality factor, k_B is the Boltzmann constant and T is temperature. The ideality factor is extracted from **Fig. S39** using the equation:

$$V_{OC}(P) = \frac{nkT}{q}\ln(P) + C$$

Where P is the incident light intensity.

The non-radiative recombination loss is equivalent to the difference between the Schockley-Queasier limit FF (FF_{SQ}) for a 1.55 eV band gap of 90.2%[21] and FF_{max}, and the transport loss is equivalent to the difference between FF_{max} and the measured device FF.

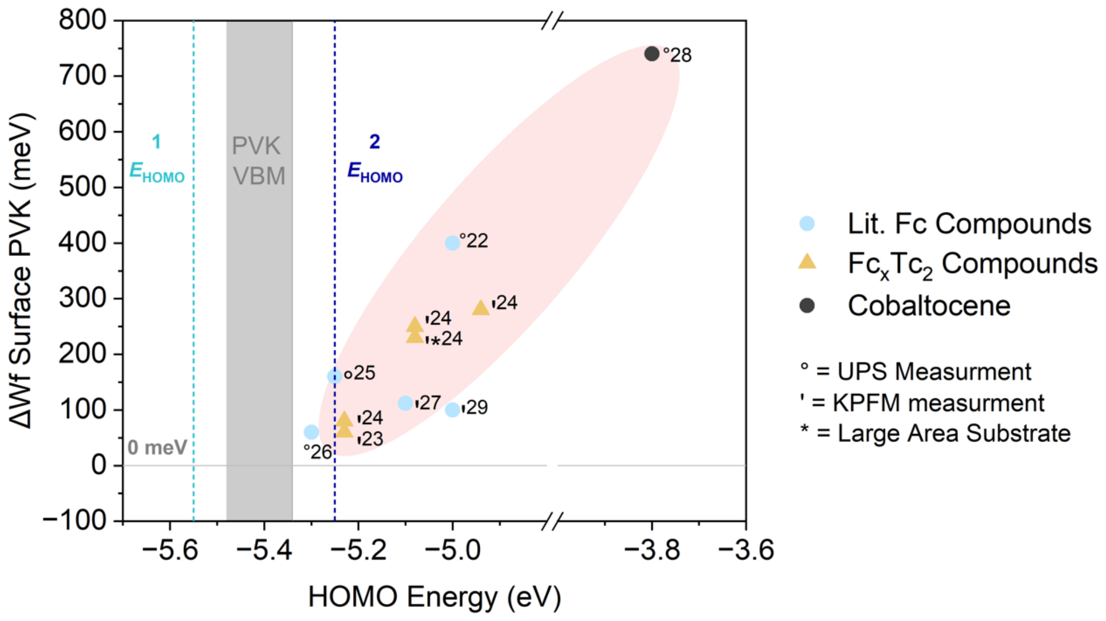

Fig. S1 Reported literature values (from refs.[22–29]) for the change in the perovskite surface work function (ΔWF) following the deposition of organometallic sandwich compounds and the respective E_{HOMO} levels of the compound used.

Table S1 Reported literature values for the change in the perovskite surface work function (ΔWF) following the deposition of organometallic sandwich compounds with the respective HOMO levels calculated according to **Supplementary Note 1**.

**Compound**	**ΔWF (meV)**	Measurement Method	**HOMO Level (eV)**
**OFFcA**	**70**[26]	UPS	**-5.3**[26]
**Fc-16 F**	**160**[25]	UPS	**-5.25**[25]
**$FcTc_2$**	**60**[23]	KPFM	**-5.23**[24]
	**80**[24]	KPFM	
**Fc**	**112**[27]	KPFM	**-5.10**[18]
**Fc_2Tc_2**	**230**[24]	KPFM	**-5.08**[24]
	**250***[24]	KPFM	
**FcDPP**	**120**[29]	KPFM	**Approx. -5.0**[30]
	**400**[22]	UPS	
**Fc_3Tc_2**	**280**[24]	KPFM	**-4.94**[24]
**Cobaltocene**	**740**[28]	UPS	**-3.8**[31,32]

* = Measured on a large area

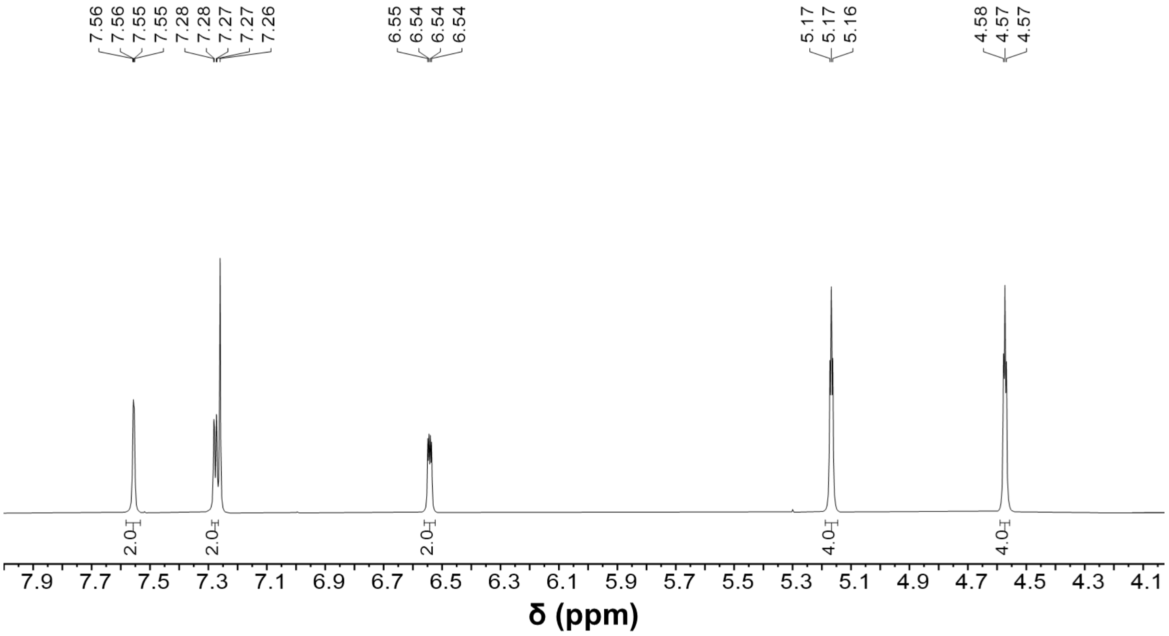

Fig. S2 ^{1}H NMR spectrum of **Compound 1** measured in $CDCl_3$.

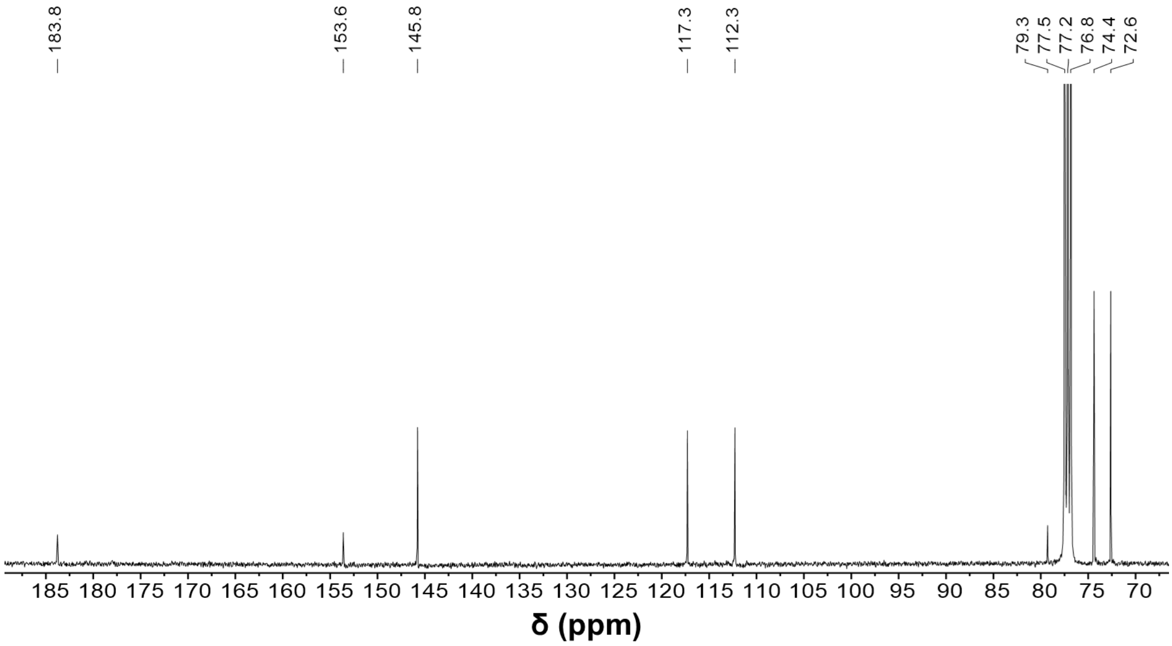

Fig. S3 $^{13}C\{^{1}H\}$ NMR spectrum of **Compound 1** measured in $CDCl_3$.

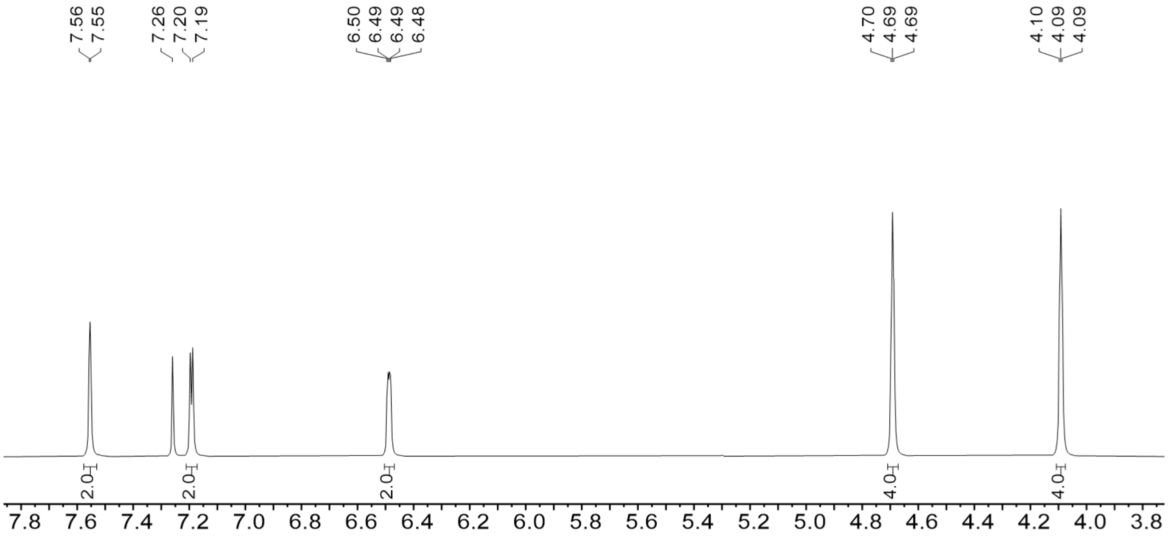

Fig. S4 ^{1}H NMR spectrum of **Compound 2** measured in $CDCl_3$.

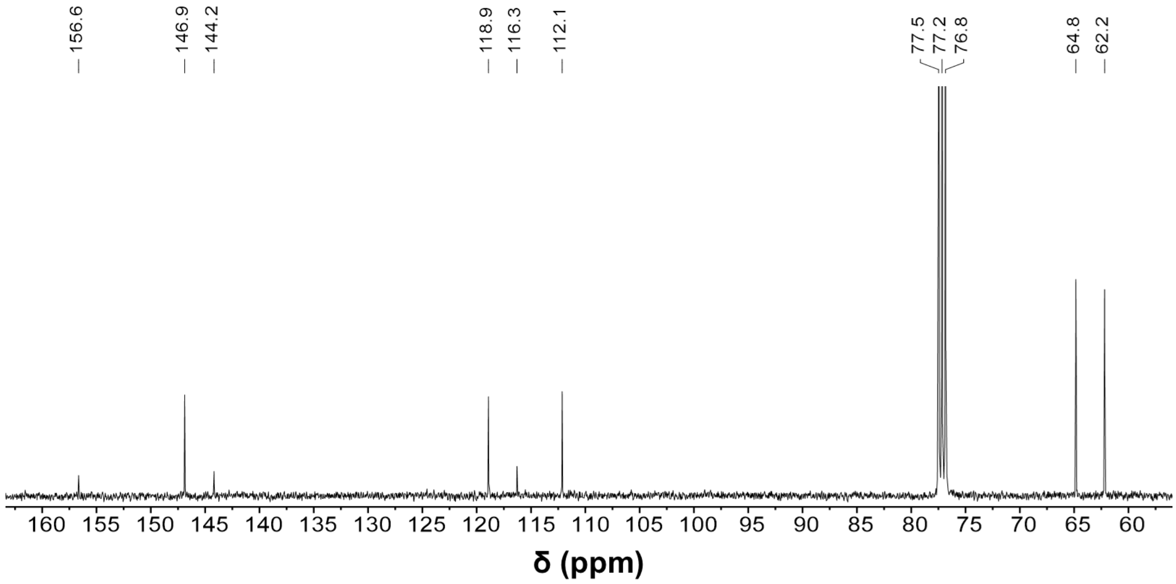

Fig. S5 $^{13}C\{^{1}H\}$ NMR spectrum of **Compound 2** measured in $CDCl_3$.

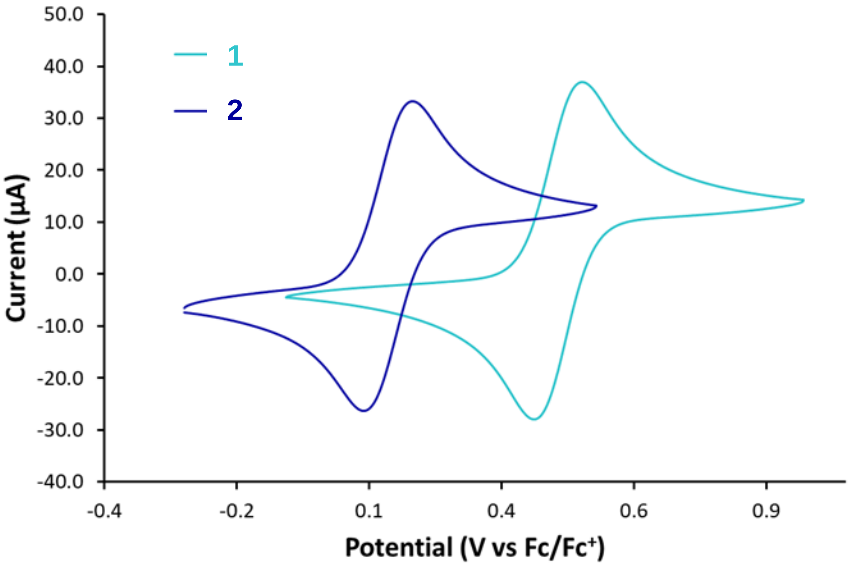

Fig. S6 Comparison cyclic voltammogram of **1** and **2** measured at 100 mV s^{-1}.

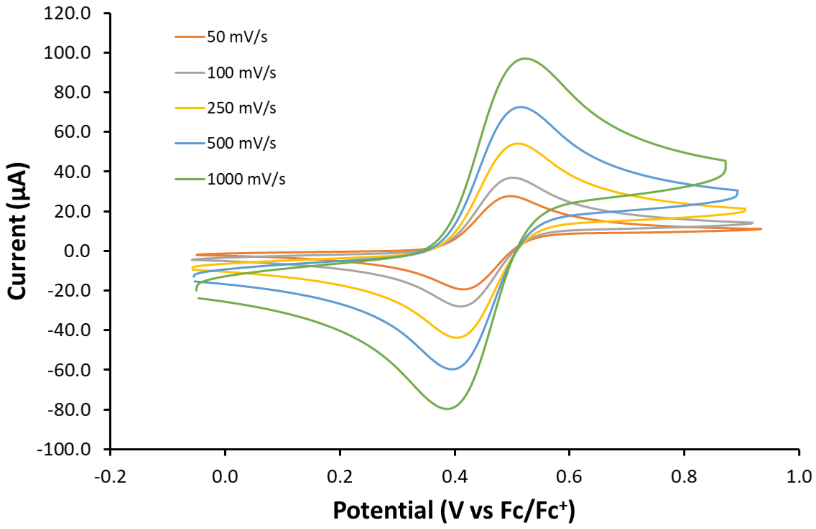

Fig. S7 Cyclic voltammogram of **1** with varying scan rates.

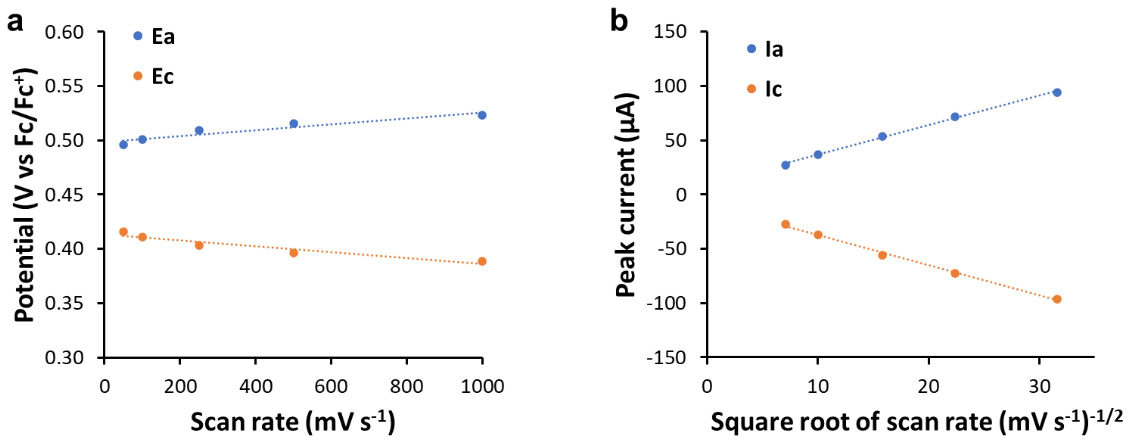

Fig. S8 Plot of potential versus scan rate (**a**) and peak current versus the square root of the scan rate (**b**) with linear fits for **1**.

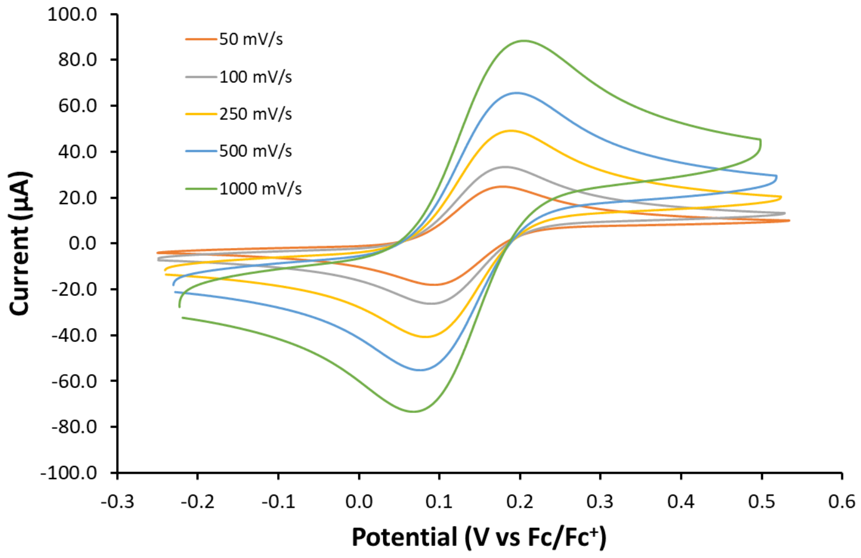

Fig. S9 Cyclic voltammogram of **2** with varying scan rates.

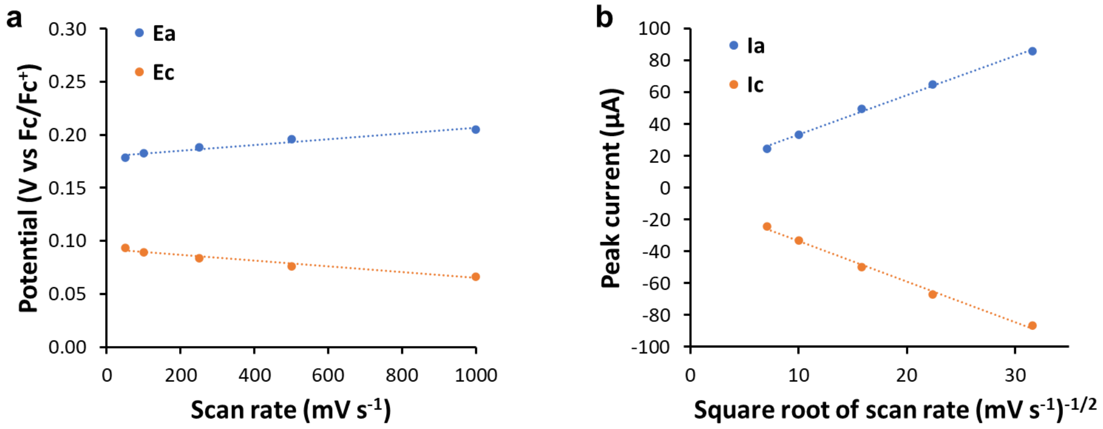

Fig. S10 Plot of potential versus scan rate (**a**) and peak current versus the square root of the scan rate (**b**) with linear fits for **2**.

Table S2 Cyclic voltammogram data (reported in mV and referenced to Fc/Fc^+ redox couple) for **1** and **2** measured at 100 mV s^{-1}. Where E_{pa} represents the voltage of the anodic peak, E_{pc} represents the voltage of the cathodic peak, $E_{1/2}$ represents the half-wave potential, and i_{pa}/i_{pc} represents the ratio of cathodic and anodic peak currents.

	E_{pa} (mV)	E_{pc} (mV)	**$E_{1/2}$** (mV)	ΔE (mV)	i_{pa}/i_{pc}
**Compound 1**	500	411	**456**	90	1.0
**Compound 2**	183	89	**136**	93	1.01

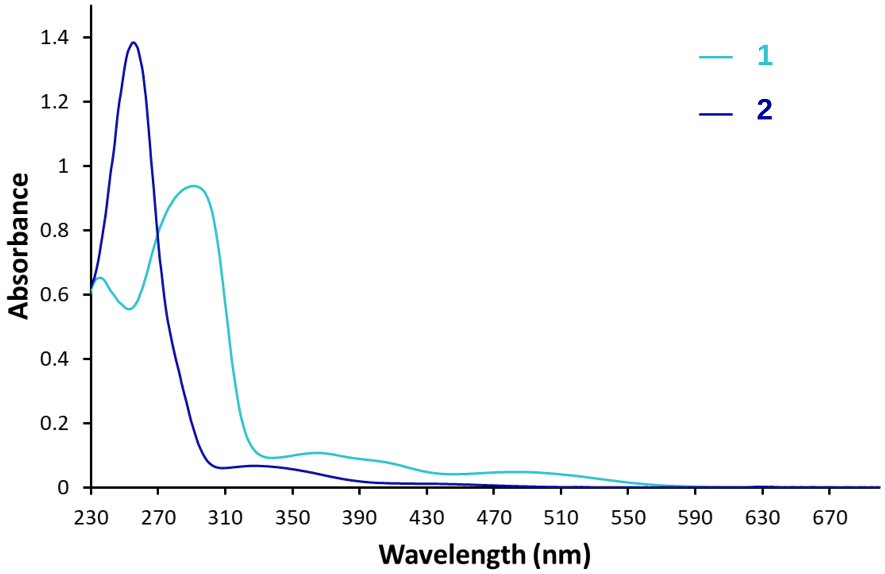

Fig. S11 UV-Vis. absorption spectra of **1** and **2** measured in CH_2Cl_2 (50 μM).

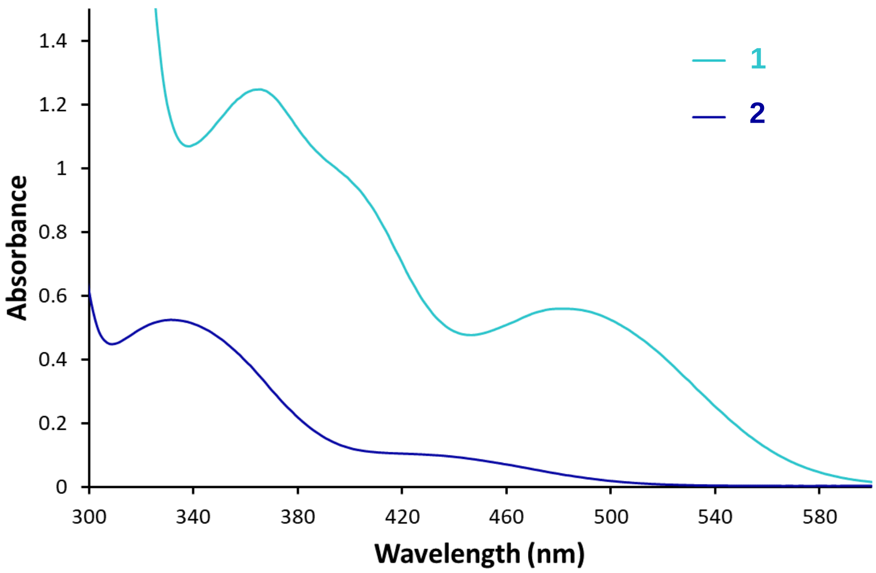

Fig. S12 UV-Vis. absorption spectra of **1** and **2** measured in CH_2Cl_2 (500 μM).

Table S3 Single-crystal X-Ray diffraction data, data collection and refinement parameters for the structures of **1** and **2**.

	**1**	**2**
**Formula**	$C_{20}H_{14}FeO_4$	$C_{20}H_{14}FeO_6$
**Solvent**	–	–
**Formula weight**	374.16	406.16
**Color, habit**	red platy	orange platy
**Temperature / K**	173	173
**Crystal system**	monoclinic	monoclinic
**Space group**	$P2_1/c$	Pc
a / Å	9.6767(6)	7.1314(2)
b / Å	11.5082(7)	20.8060(6)
c / Å	6.7495(6)	11.1871(4)
**α / deg**	90	90
**β / deg**	92.758(8)	108.043(4)
**γ / deg**	90	90
V / Å^3	750.76(9)	1578.27(9)
Z	2	4[c]
D_c / g cm^{-3}	1.655	1.709
**Radiation used**	Cu Kα	Mo Kα
**μ / mm^{-1}**	8.264	0.994
**No. of unique reflns**		
**measured (R_{int})**	2353 (0.0317)	11063 (0.0295)
**obs, $\lvert F_o \rvert > 4\sigma(\lvert F_o \rvert)$**	1125	4321
**Completeness (%)[a]**	97.3	99.6
**No. of variables**	118	493
R_1(obs), wR_2(all)[b]	0.0626, 0.1749	0.0348, 0.0734
**CCDC code**	2390047	2390048

[a] Completeness to 0.84 Å resolution. [b] $R_1 = \Sigma\lvert\lvert F_o\rvert - \lvert F_c\rvert\rvert/\Sigma\lvert F_o\rvert$; $wR_2 = \{\Sigma[w(F_o^2 - F_c^2)^2] / \Sigma[w(F_o^2)^2]\}^{1/2}$; $w^{-1} = \sigma^2(F_o^2) + (aP)^2 + bP$. [c] There are two crystallographically independent molecules.

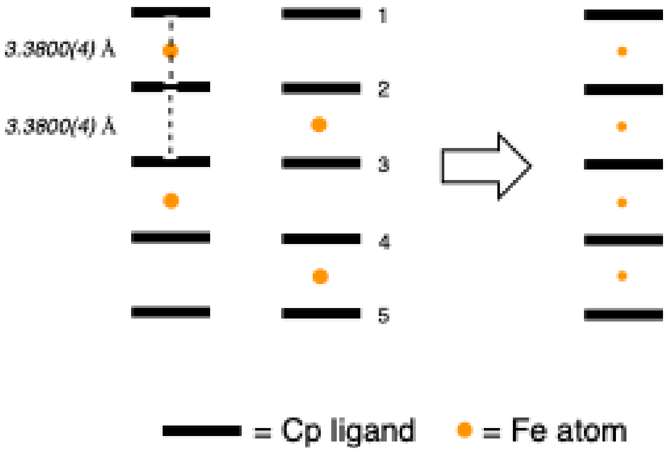

Fig. S13: Visual representation of the packing of the disordered ferrocene units in **1**.

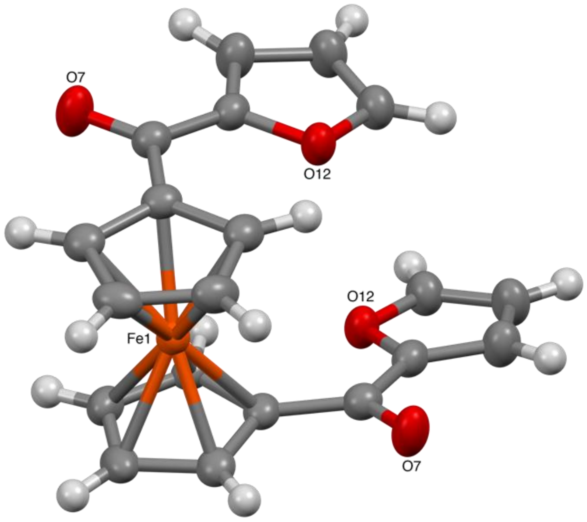

Figure S14: The structure of **1** (50% probability ellipsoids).

a

b

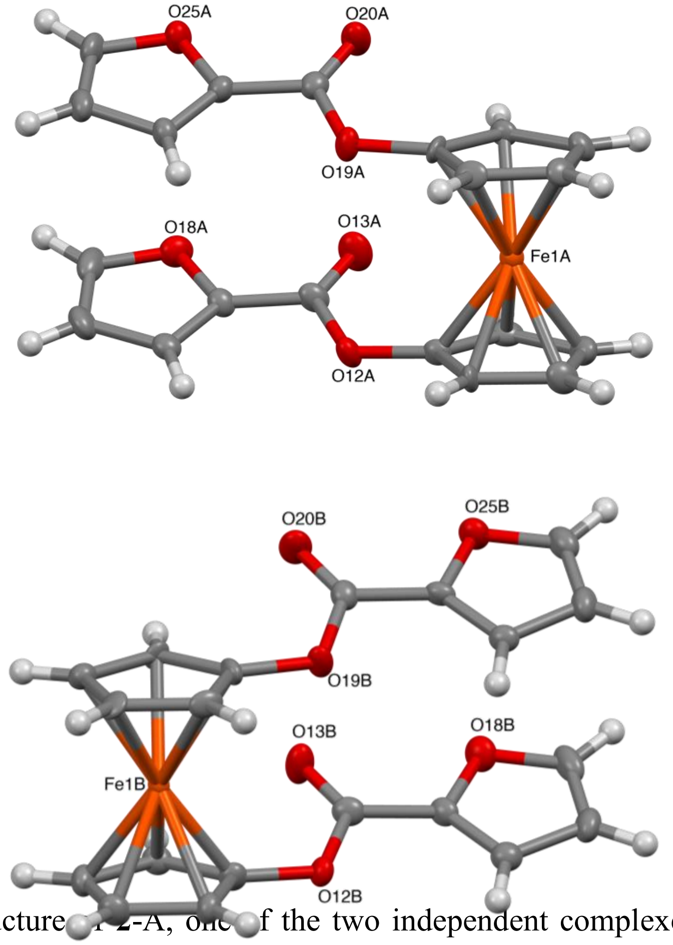

Figure S15: **a**, The structure of **2**-A, one of the two independent complexes present in the crystal of **2** (50% probability ellipsoids). **b**, The structure of **2**-B, one of the two independent complexes present in the crystal of **2** (50% probability ellipsoids).

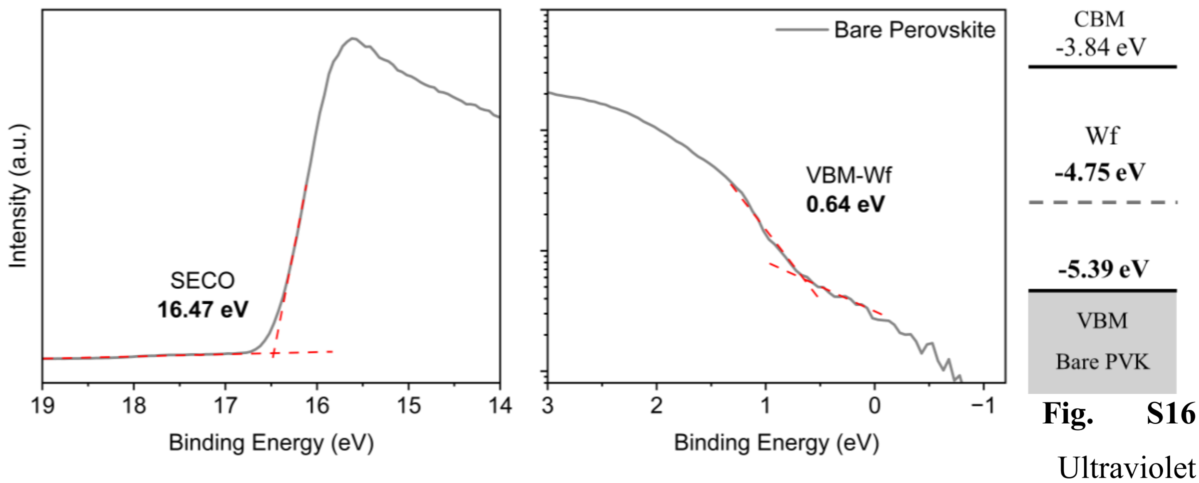

Fig. S16 Ultraviolet photoelectron spectra and resulting energy level diagram of the bare perovskite surface. Left: secondary electron cutoff region (SECO); Right: valance band region plotted on a logarithmic scale.

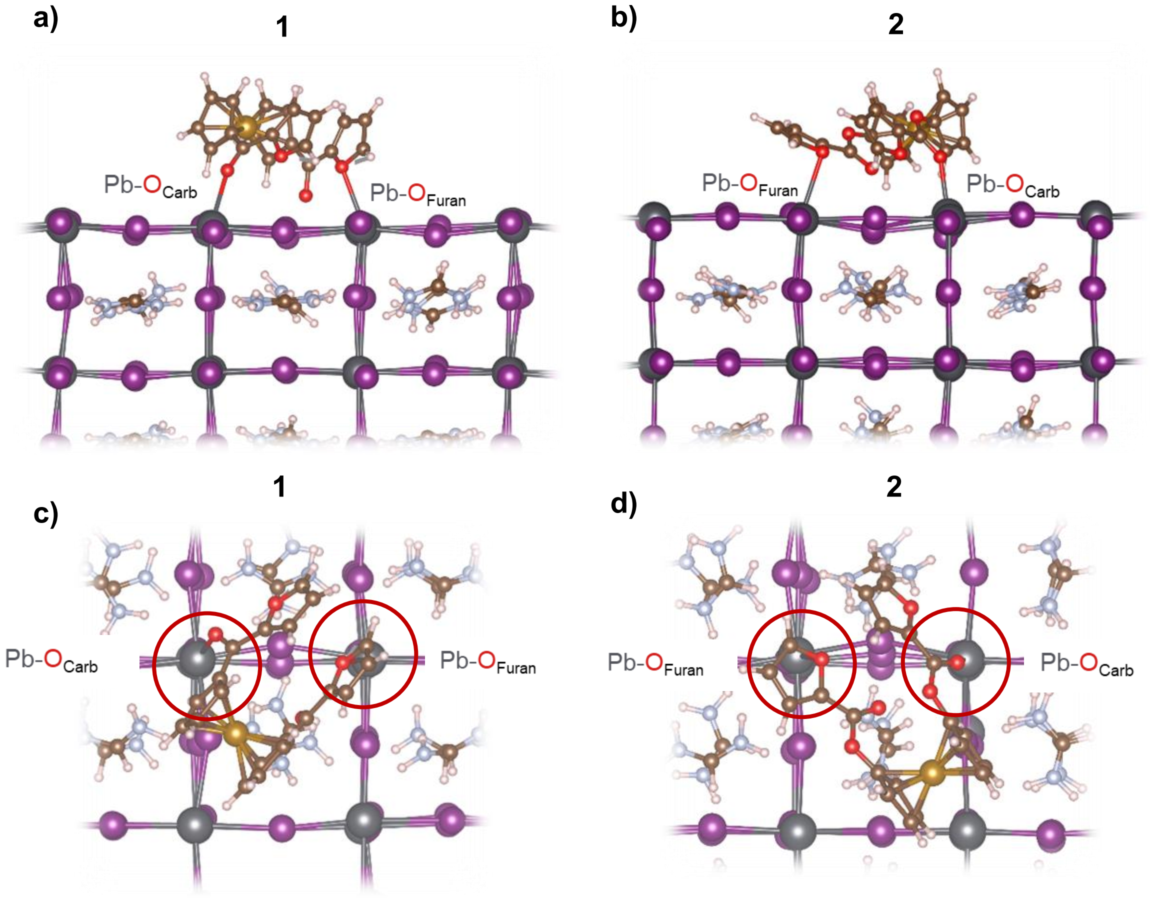

Fig. S17 (a), (b) Side-view and (c), (d) top-view of structural simulation results revealing the interactions between **1** and **2** in the syn- conformation on the perovskite surface. Note the bidentate surface binding enabled through furan oxygens.

Table S4 Predicted Pb-O bond lengths for **1** and **2** passivated $FAPbI_3$ surface in Anti- and Syn-conformations obtained using DFT.

	**Compound 1**		**Compound 2**	
Bond	Anti- (Å)	Syn- (Å)	Anti- (Å)	Syn- (Å)
**1Pb-O$_{\mathbf{Carb}}$**	2.41	2.64	2.58	2.50
**2Pb-O$_{\mathbf{Carb}}$**	2.46	-	2.70	-
**Pb-O$_{\mathbf{Furan}}$**	-	2.84	-	3.00

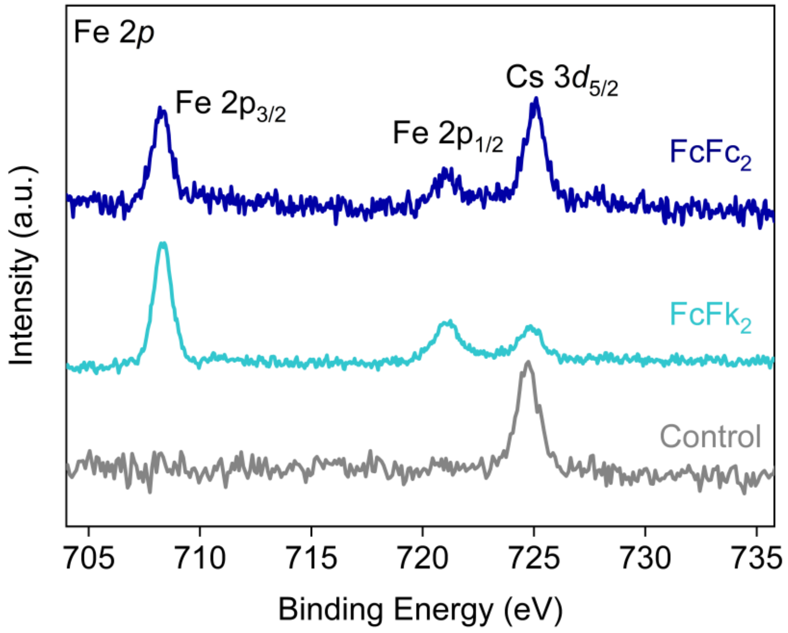

Fig. S18 XPS spectra showing the Fe 2p region of triple-cation perovskite samples treated with **1** and **2**. Note the overlap of the Cs $3d_{5/2}$ and Fe $2p_{1/2}$ signals. Also note the lower intensity of the Fe 2p peaks in sample **2** compared to sample **1** and the potential presence of low-intensity Fe^{3+} peaks at higher binding energies in sample **2**.

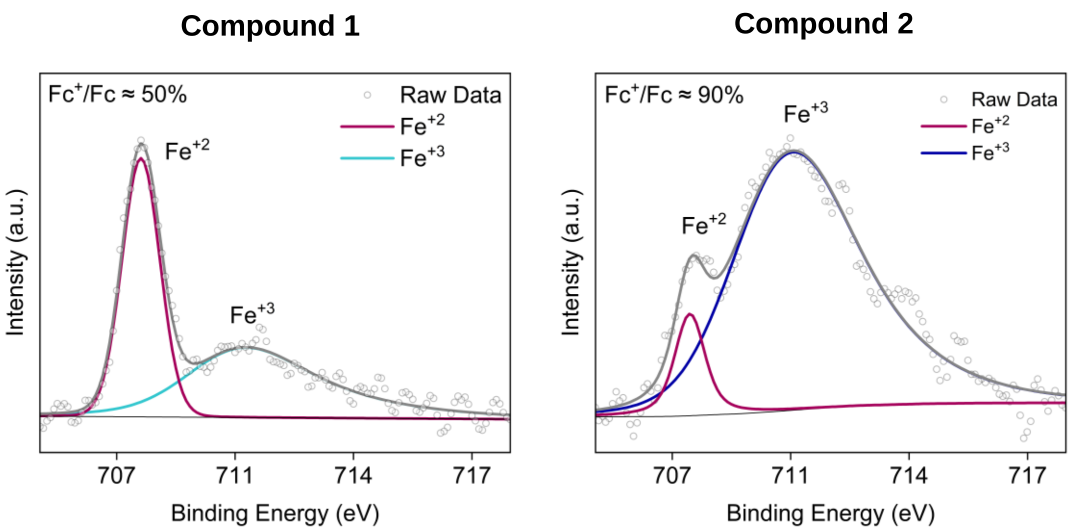

Fig. S19 XPS spectra showing the Fe 2p region of PbI_2 thin film samples treated with **1** and **2** fitted according to the two oxidation states of Fe present.

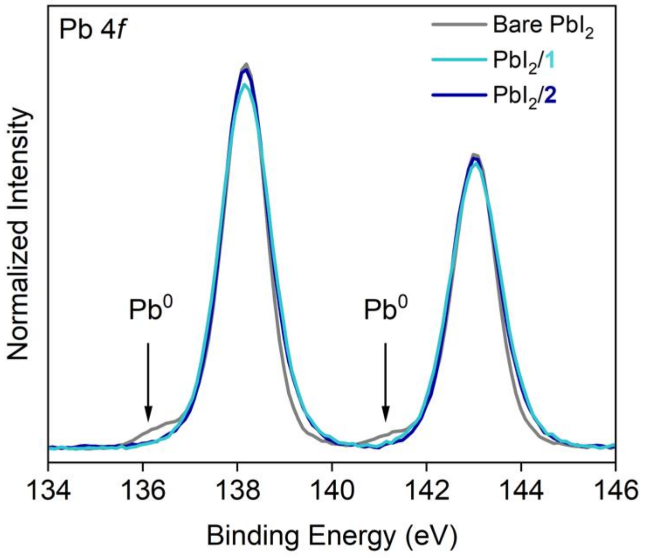

Fig. S20 XPS spectra showing the Pb 4f region of PbI_2 thin film samples treated with **1** and **2**. The peaks at lower binding energies are widely attributed to Pb^0 and are clearly suppressed in Fc-passivated samples, consistent with previous observations.[25,33,34]

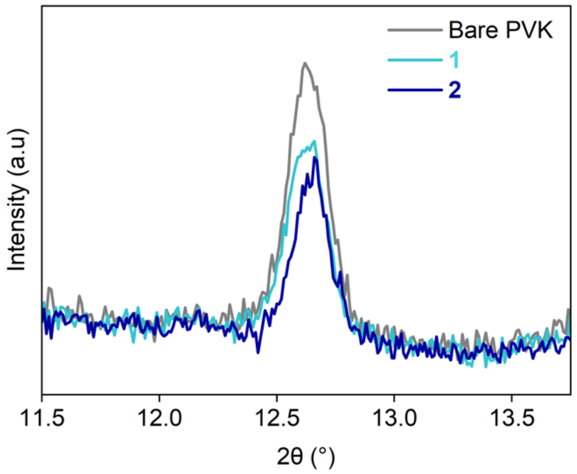

Fig. S21 XRD spectra showing the effect of **1** and **2** deposition on the PbI_2 peak intensity centered at 12.7° in perovskite films.

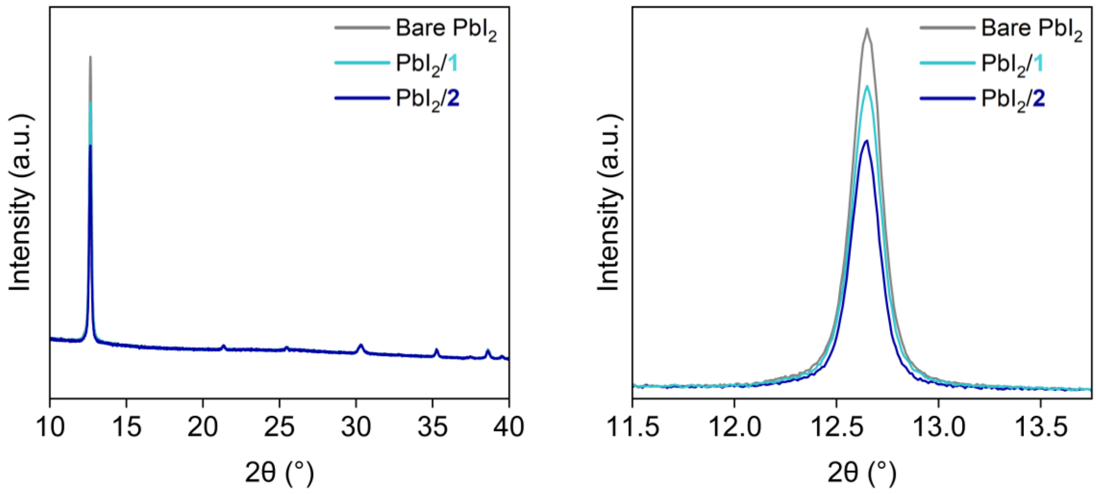

Fig. S22 Left: XRD spectra showing the effect of **1** and **2** deposition on PbI_2 thin films; Right: zoom of the decrease in the PbI_2 peak intensity centered at 12.7°.

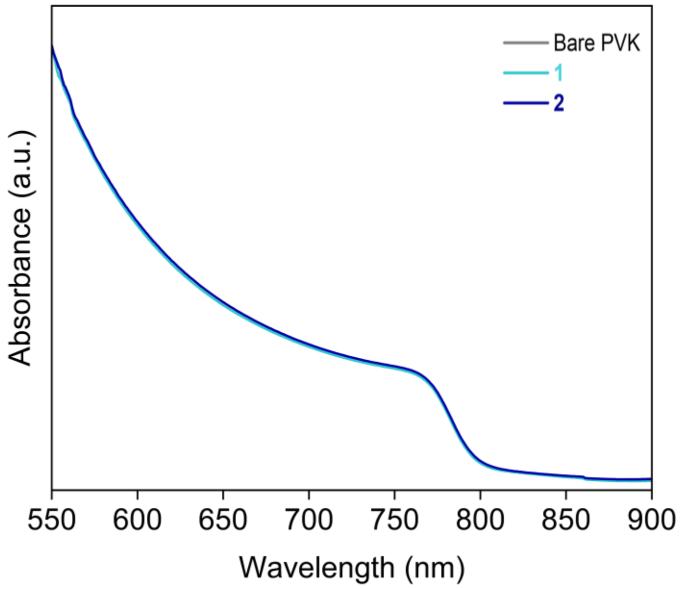

Fig. S23 UV-Vis. absorbance spectra of perovskite thin films treated with Fc compounds.

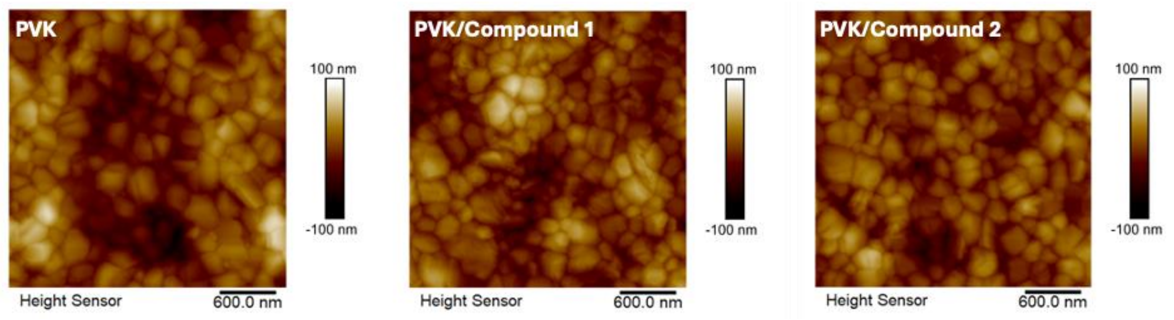

Fig. S24 AFM images of perovskite thin films treated with Fc compounds.

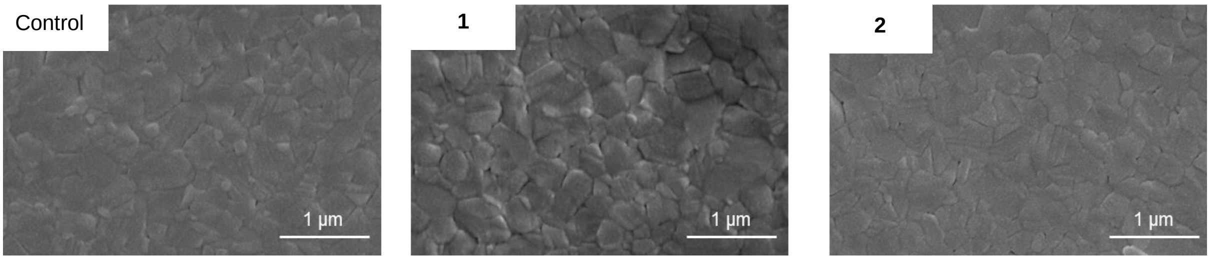

Fig. S25 SEM images of perovskite thin films treated with Fc compounds.

Table S5. TRPL lifetimes obtained by biexponential fitting of transient PL spectra shown in **Figure 2f**. Average lifetimes were calculated using the following equation $\tau_{avg} = \frac{A_1\tau_1{}^2 + A_2\tau_2{}^2}{(A_1\tau_1 + A_2\tau_2)}$ and are tabulated below.

Sample	A_1	τ_1 (ns)	A_2	τ_2 (ns)	$\boldsymbol{\tau_{avg}}$ **(ns)**
**Control**	0.40	288.8	0.47	1201.2	**1046.2**
**Compound 1**	0.36	292.2	0.49	1318.8	**1175.1**
**Compound 2**	0.34	456.3	0.51	1715.4	**1525.8**

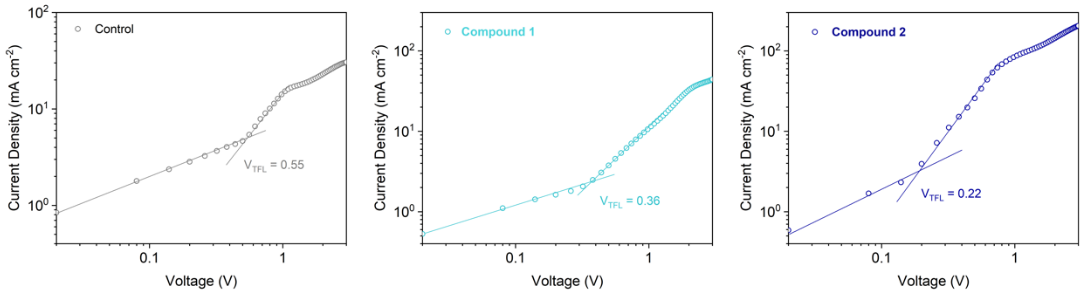

Fig. S26 SCLC measurements of electron-only devices having an ITO/SnO_2/Perovskite/Fc-interlayer/C_{60}/BCP/Ag architecture with the different Fc interlayers.

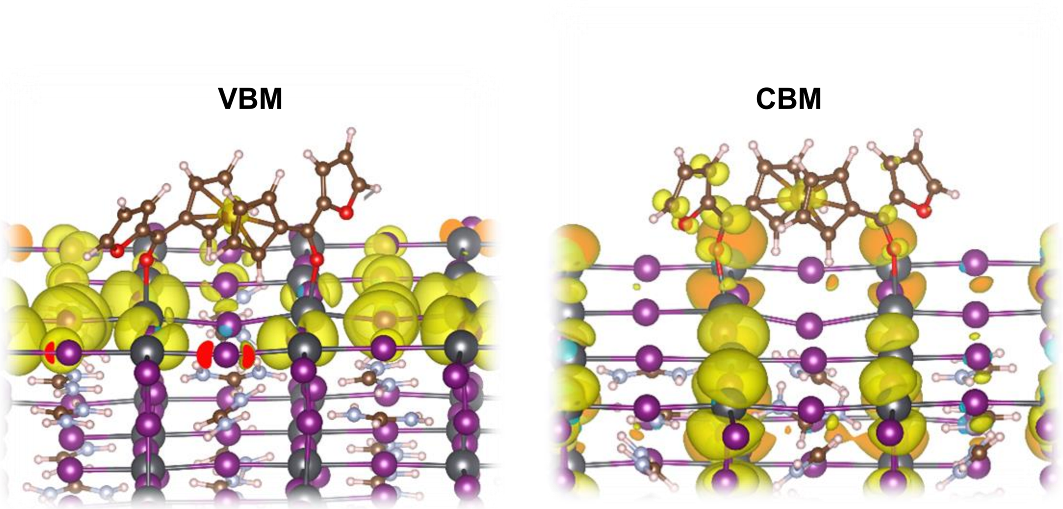

Fig. S27 Predicted spatial charge distribution of the valance and conduction bands of the (001) $FAPbI_3$ surface passivated with **1** bound in the bidentate anti-conformation.

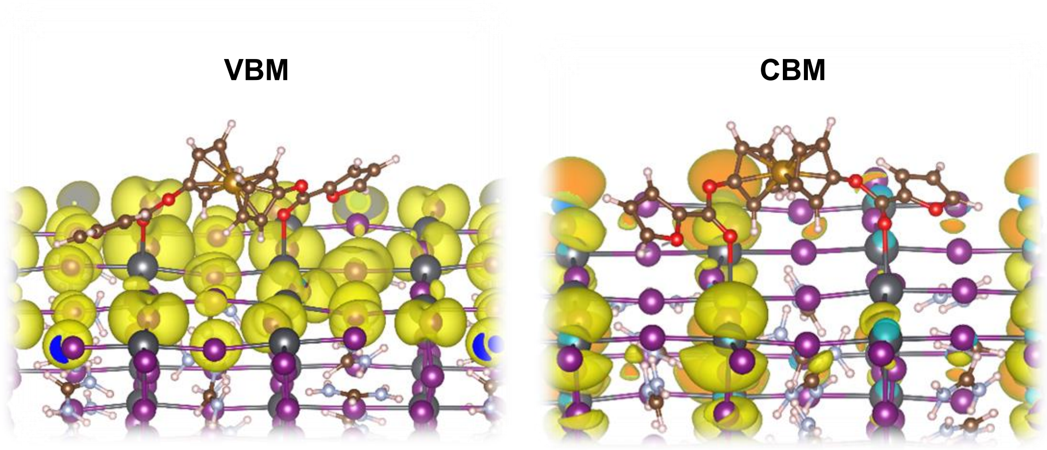

Fig. S28 Predicted spatial charge distribution of the valance and conduction bands of the (001) $FAPbI_3$ surface passivated with **2** bound in the bidentate anti-conformation.

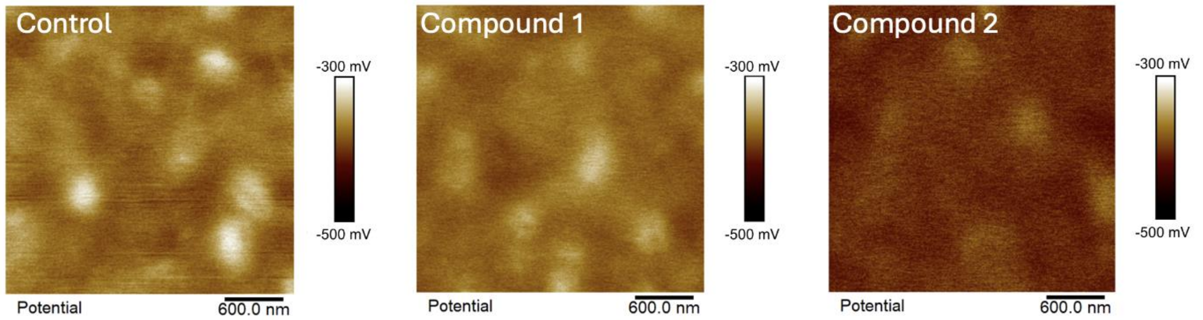

Fig. S29 KPFM mapping images of the contact potential difference (CPD) of perovskite samples treated with Fc molecules. WF values were obtained using the equation CPD = ϕ^{TIP}-ϕ^{SAMPLE} calibrating the tip using an Au reference with a work function of -5.1 eV.

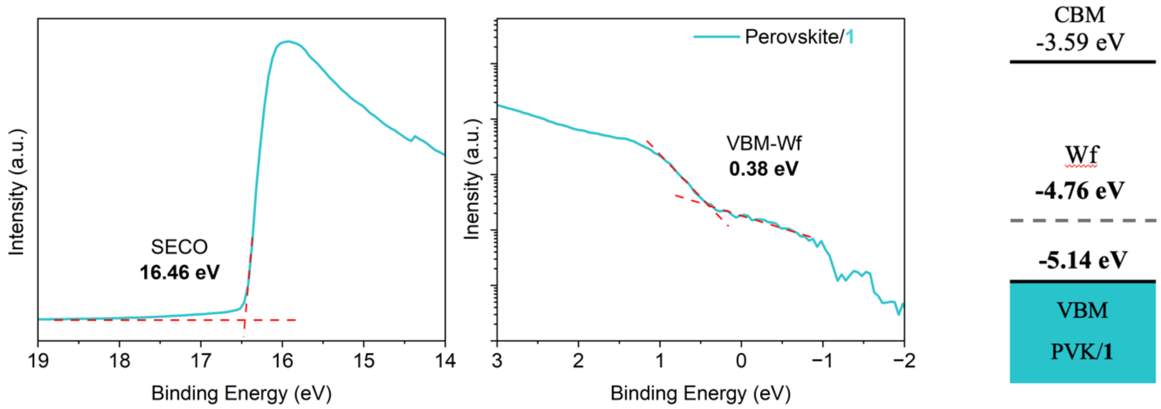

Fig. S30 Ultraviolet photoelectron spectra and resulting energy level diagram of the perovskite surface treated with compound **1**. Left: secondary electron cutoff region (work function); Right: valance band region plotted on a logarithmic scale.

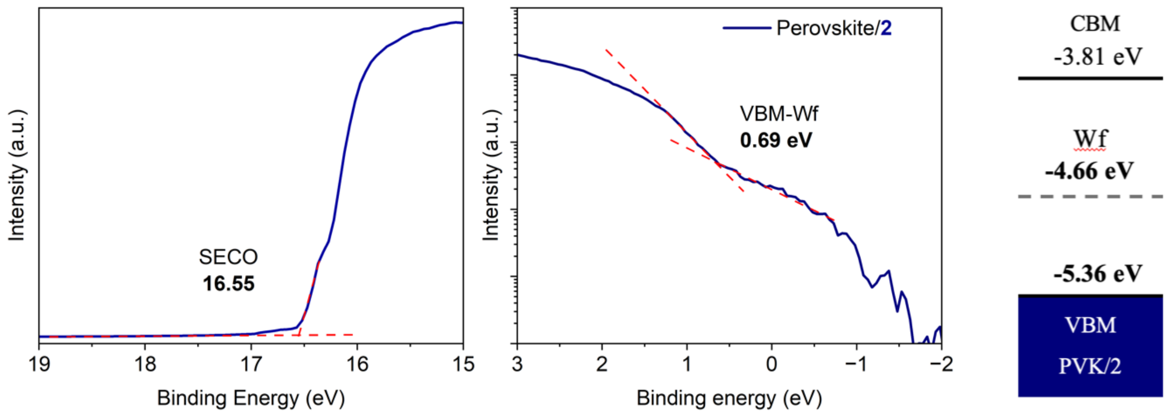

Fig. S31 Ultraviolet photoelectron spectra and resulting energy level diagram of the perovskite surface treated with compound **2**. Left: secondary electron cut-off region (work function); Right: valance band region plotted on a logarithmic scale.

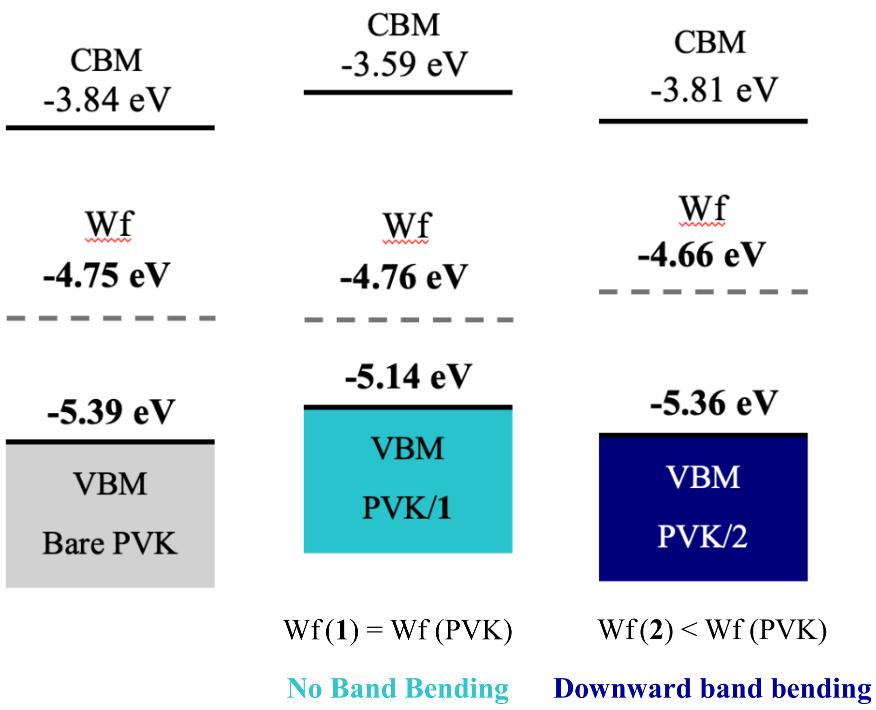

Fig. S32 VBM, CBM (obtained by adding the perovskite band gap, 1.55 eV) and WF values extracted from the UPS data shown in **Fig S16**, **S30** and **S31**.

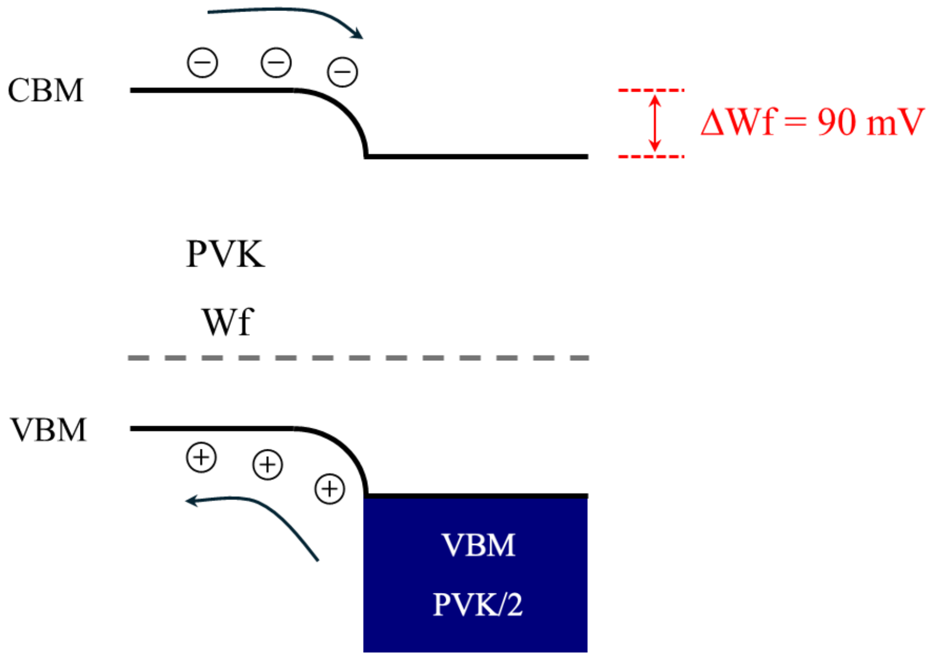

Fig. S33 Sketch of the band bending at the perovskite/**2** interface caused by the change in surface Wf following the deposition of compound **2**.

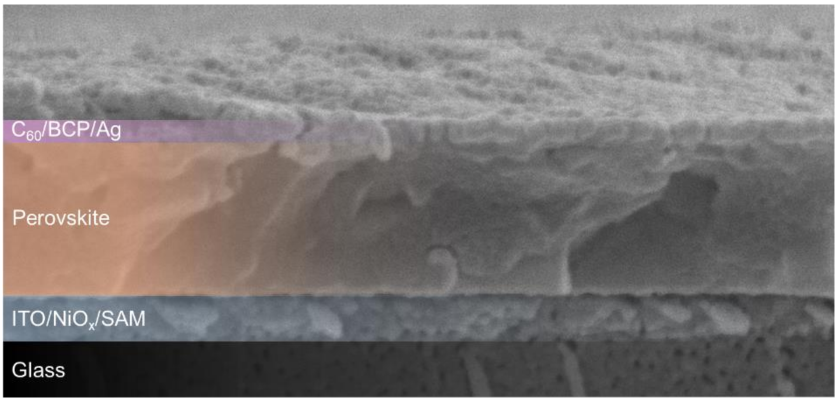

Fig. S34 Cross-sectional scanning electron-microscopy image of a representative device having the ITO/NiO_x/SAM/Perovskite/C_{60}/BCP/Ag structure.

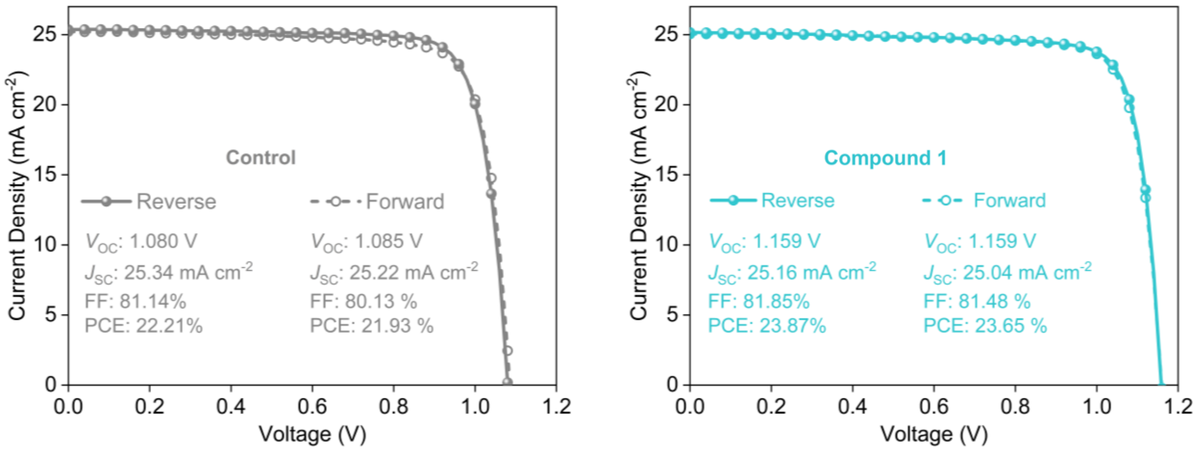

Fig. S35 J-V curves of champion control and compound **1** modified devices.

Table S6 Photovoltaic parameters extracted from champion control, **1** and **2** treated devices.

Sample	J_{SC} (mA cm^{-2})	V_{OC} (V)	FF (%)	PCE (%)
Control	25.34	1.080	81.14	22.21
**1**	25.16	1.159	81.85	23.87
**2**	25.36	1.183	83.86	25.16

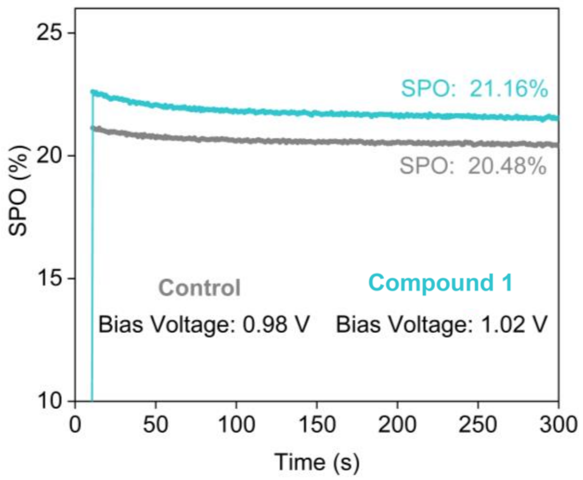

Fig. S36 SPO of control and **1** functionalized devices.

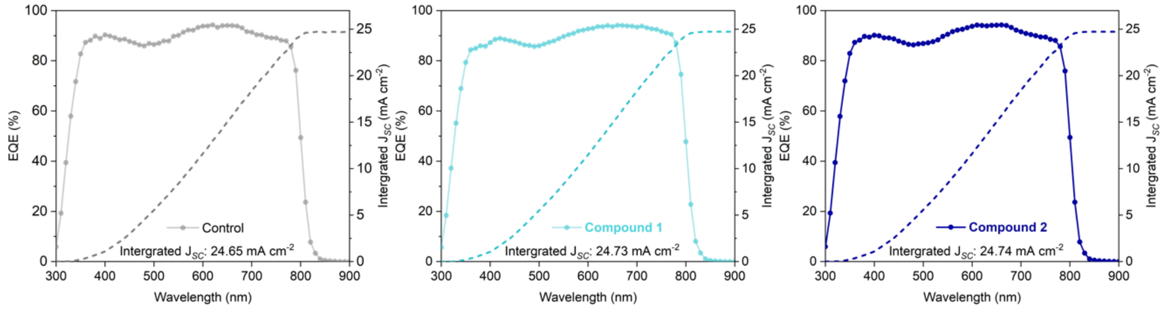

Fig. S37 EQE spectra of control, **1** and **2** functionalized devices with respective J_{SC} values obtained by integrating the spectrum.

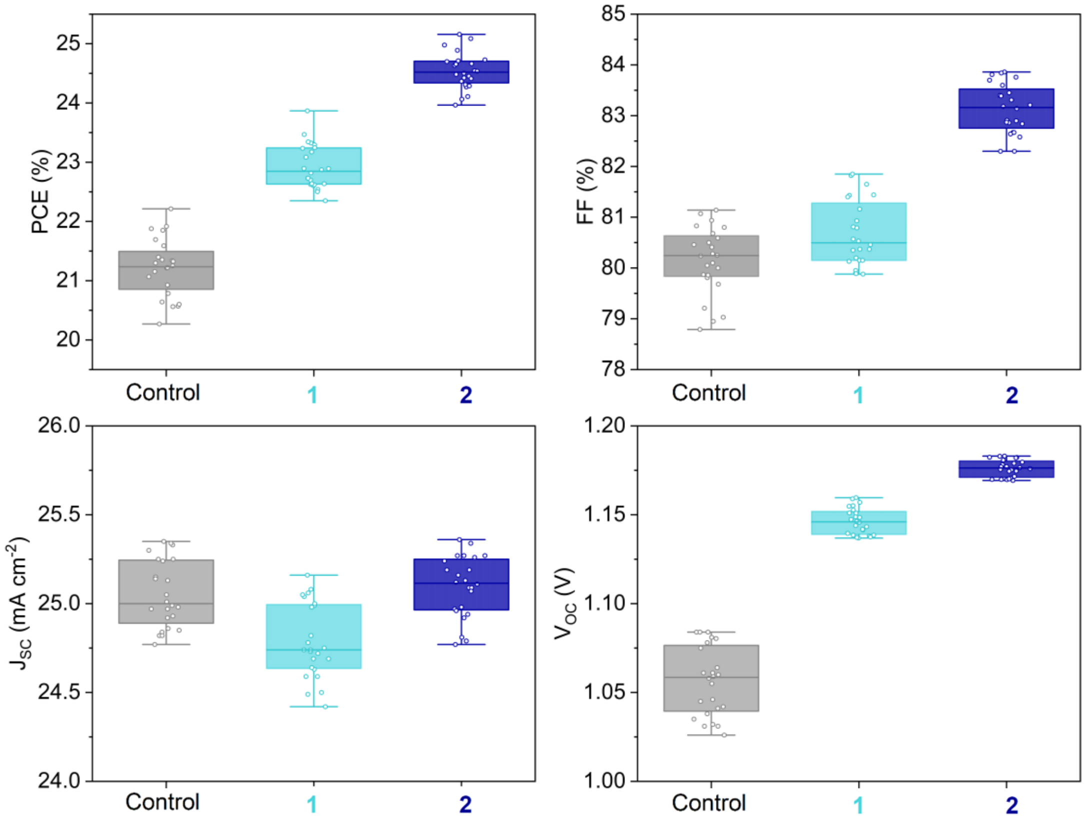

Fig. S38 Statistics of photovoltaic parameters obtained from the J-V characteristic of 24 control devices, and devices modified with **1** and **2**.

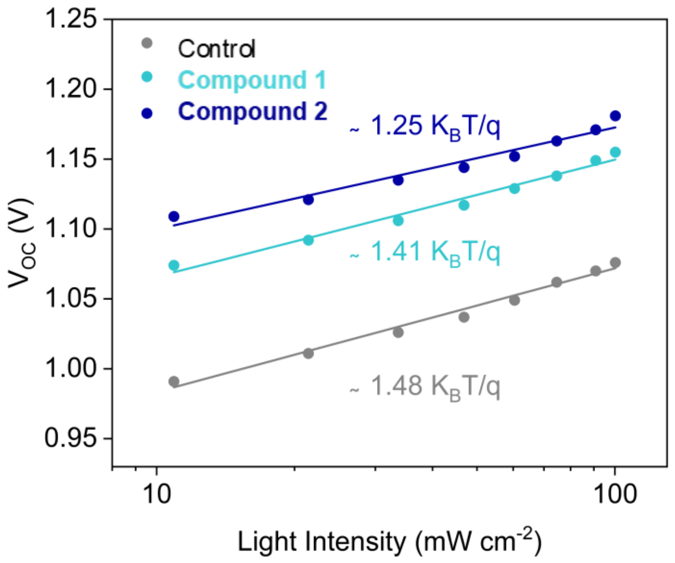

Fig. S39 Fitted intensity-dependent V_{OC} measurements with ideality factors calculated according to **Note S3**.

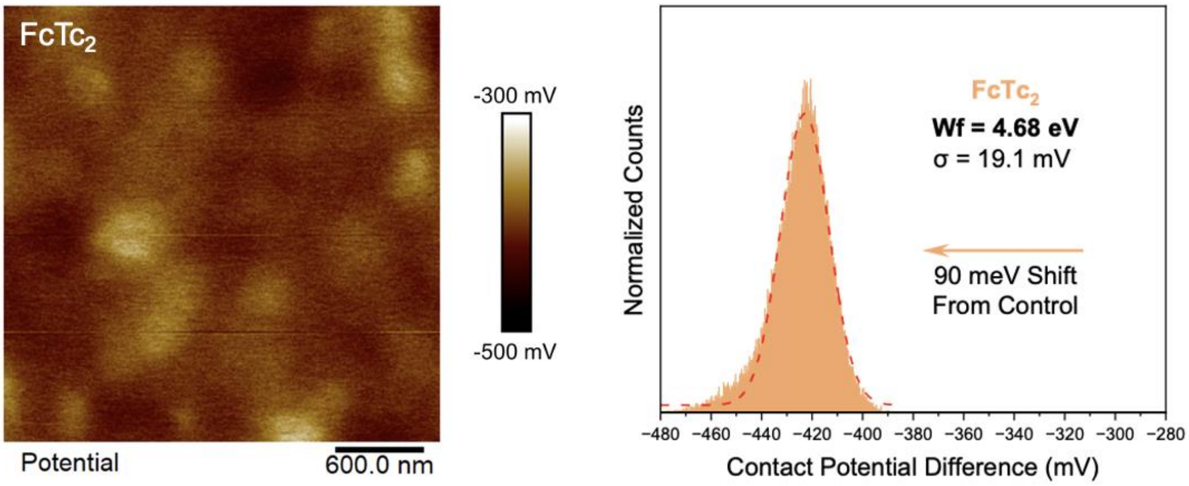

Fig. S40 KPFM mapping image of the contact potential difference (CPD) of a perovskite film treated with $FcTc_2$ alongside the statistical distribution of the CPD. The WF value was obtained using the equation CPD = ϕ^{TIP}-ϕ^{SAMPLE} calibrating the tip using an Au reference with a work function of -5.1 eV.

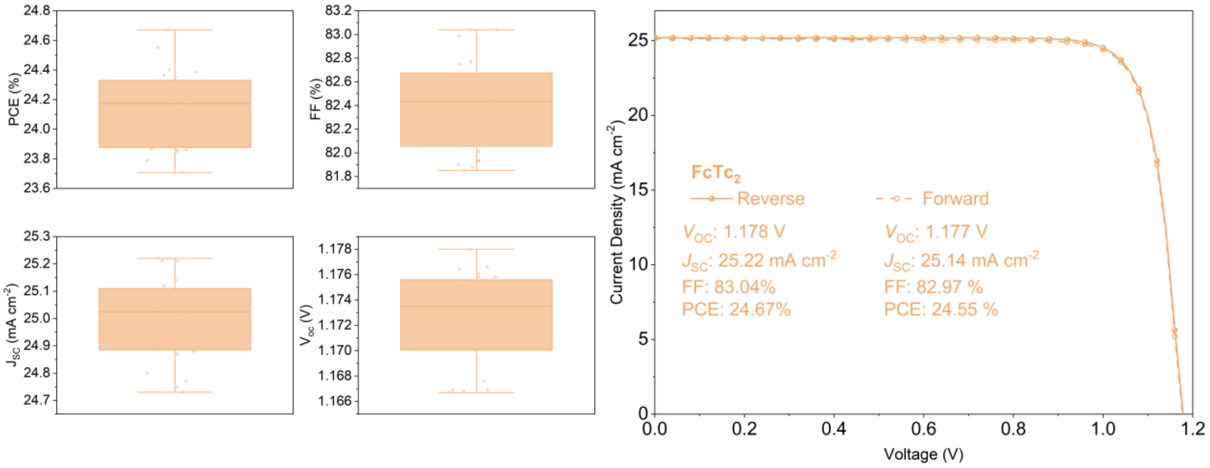

Fig. S41 Statistics of photovoltaic parameters obtained from the J-V characteristic of 24 PSCs featuring $FcTc_2$ interlayers and champion $FcTc_2$ treated device performance in forward and reverse scans.

References

[1] M. Roemer, C. A. Nijhuis, *Dalton Trans.* 2014, *43*, 11815–11818.

[2] A. A. O. Sarhan, M. S. Ibrahim, M. M. Kamal, K. Mitobe, T. Izumi, *Monatsh Chem* 2009, *140*, 315–323.

[3] G. Kresse, *Journal of Non-Crystalline Solids* 1995, *192–193*, 222–229.

[4] P. E. Blöchl, *Phys. Rev. B* 1994, *50*, 17953–17979.

[5] J. P. Perdew, K. Burke, M. Ernzerhof, *Phys. Rev. Lett.* 1996, *77*, 3865–3868.

[6] S. Grimme, *Journal of Computational Chemistry* 2006, *27*, 1787–1799.

[7] S. Grimme, J. Antony, S. Ehrlich, H. Krieg, *The Journal of Chemical Physics* 2010, *132*, 154104.

[8] J. Moellmann, S. Grimme, *J. Phys. Chem. C* 2014, *118*, 7615–7621.

[9] O. V. Dolomanov, L. J. Bourhis, R. J. Gildea, J. a. K. Howard, H. Puschmann, *J Appl Cryst* 2009, *42*, 339–341.

[10] G. M. Sheldrick, *Acta Cryst C* 2015, *71*, 3–8.

[11] T. Gruene, H. W. Hahn, A. V. Luebben, F. Meilleur, G. M. Sheldrick, *J Appl Cryst* 2014, *47*, 462–466.

[12] N. Fairley, V. Fernandez, M. Richard-Plouet, C. Guillot-Deudon, J. Walton, E. Smith, D. Flahaut, M. Greiner, M. Biesinger, S. Tougaard, D. Morgan, J. Baltrusaitis, *Applied Surface Science Advances* 2021, *5*, 100112.

[13] G. Roy, R. Gupta, S. Ranjan Sahoo, S. Saha, D. Asthana, P. Chandra Mondal, *Coordination Chemistry Reviews* 2022, *473*, 214816.

[14] L. Fabbrizzi, *ChemTexts* 2020, *6*, 22.

[15] G. P. Kissling, B. Ruhstaller, K. P. Pernstich, *Organic Electronics* 2023, *122*, 106888.

[16] G. Gritzner, J. Kuta, *Pure and Applied Chemistry* 1984, *56*, 461–466.

[17] N. Elgrishi, K. J. Rountree, B. D. McCarthy, E. S. Rountree, T. T. Eisenhart, J. L. Dempsey, *J. Chem. Educ.* 2018, *95*, 197–206.

[18] C. M. Cardona, W. Li, A. E. Kaifer, D. Stockdale, G. C. Bazan, *Advanced Materials* 2011, *23*, 2367–2371.

[19] W. N. Hansen, G. J. Hansen, *Phys. Rev. A* 1987, *36*, 1396–1402.

[20] A. J. Bard, L. R. Faulkner, *Electrochemical Methods: Fundamentals and Applications*, Wiley, New York Weinheim, 2001.

[21] N. K. Elumalai, M. A. Mahmud, D. Wang, A. Uddin, *Energies* 2016, *9*, 861.

[22] K. Hu, J. Peng, Q. Wang, L. Deng, C. Deng, M. Xu, Z. Zhang, H. Ren, C. Yang, J. Chen, H. Yu, *Solar Energy Materials and Solar Cells* 2024, *272*, 112936.

[23] Z. Li, B. Li, X. Wu, S. A. Sheppard, S. Zhang, D. Gao, N. J. Long, Z. Zhu, *Science* 2022, *376*, 416–420.

[24] B. Li, D. Gao, S. A. Sheppard, W. D. J. Tremlett, Q. Liu, Z. Li, A. J. P. White, R. K. Brown, X. Sun, J. Gong, S. Li, S. Zhang, X. Wu, D. Zhao, C. Zhang, Y. Wang, X. C. Zeng, Z. Zhu, N. J. Long, *J. Am. Chem. Soc.* 2024, *146*, 13391–13398.

[25] B. Hu, J. Zhang, Y. Yang, Y. Dong, J. Wang, W. Wang, K. Lin, D. Xia, R. Fan, *Nano Energy* 2023, *118*, 109022.

[26] B. Hu, J. Zhang, Y. Yang, Y. Dong, J. Wang, W. Wang, X. Zhang, K. Lin, D. Xia, *Journal of Energy Chemistry* 2024, *98*, 645–655.

[27] T. Webb, X. Liu, R. J. E. Westbrook, S. Kern, M. T. Sajjad, S. Jenatsch, K. D. G. I. Jayawardena, W. H. K. Perera, I. P. Marko, S. Sathasivam, B. Li, M. Yavari, D. J. Scurr, M. R. Alexander, T. J. Macdonald, S. A. Haque, S. J. Sweeney, W. Zhang, *Advanced Energy Materials* 2022, *12*, 2200666.

[28] E. E. Perry, J. G. Labram, N. R. Venkatesan, H. Nakayama, M. L. Chabinyc, *Advanced Electronic Materials* 2018, *4*, 1800087.

[29] J. Guo, B. Wang, J. Min, J. Shi, Y. Wang, X. Ling, Y. Shi, I. Ullah, D. Chu, W. Ma, J. Yuan, *ACS Nano* 2024, *18*, 19865–19874.
[30] B. Corain, B. Longato, G. Favero, D. Ajò, G. Pilloni, U. Russo, F. R. Kreissl, *Inorganica Chimica Acta* 1989, *157*, 259–266.
[31] S. P. Gubin, S. A. Smirnova, L. I. Denisovich, *Journal of Organometallic Chemistry* 1971, *30*, 257–265.
[32] G. E. McManis, M. N. Golovin, M. J. Weaver, *J. Phys. Chem.* 1986, *90*, 6563–6570.
[33] Q. Chang, F. Wang, W. Xu, A. Wang, Y. Liu, J. Wang, Y. Yun, S. Gao, K. Xiao, L. Zhang, L. Wang, J. Wang, W. Huang, T. Qin, *Angewandte Chemie International Edition* 2021, *60*, 25567–25574.
[34] D. Ou, W. Ye, M.-H. Shang, J. Tu, J. Zheng, L. Wang, W. Yang, Z. Du, Z. Yang, *ACS Appl. Mater. Interfaces* 2023, *15*, 42697–42705.
